# An integrative review of systematic reviews related to the management of breathlessness in respiratory illnesses

**DOI:** 10.1186/1471-2466-10-63

**Published:** 2010-12-09

**Authors:** Chris D Bailey, Richard Wagland, Rasha Dabbour, Ann Caress, Jaclyn Smith, Alex Molassiotis

**Affiliations:** 1 Faculty of Health Sciences, University of Southampton, Highfield, Southampton, SO17 1BJ, UK; 2 School of Nursing, Midwifery & Social Work, University of Manchester, Manchester M13 9PL, UK; 3 Department of Translational Medicine, University of Manchester, Manchester, UK & Johns Hopkins Asthma and Allergy Center, Boston, USA

## Abstract

**Background:**

Breathlessness is a debilitating and distressing symptom in a wide variety of diseases and still a difficult symptom to manage. An integrative review of systematic reviews of non-pharmacological and pharmacological interventions for breathlessness in non-malignant disease was undertaken to identify the current state of clinical understanding of the management of breathlessness and highlight promising interventions that merit further investigation.

**Methods:**

Systematic reviews were identified via electronic databases between July 2007 and September 2009. Reviews were included within the study if they reported research on adult participants using either a measure of breathlessness or some other measure of respiratory symptoms.

**Results:**

In total 219 systematic reviews were identified and 153 included within the final review, of these 59 addressed non-pharmacological interventions and 94 addressed pharmacological interventions. The reviews covered in excess of 2000 trials. The majority of systematic reviews were conducted on interventions for asthma and COPD, and mainly focussed upon a small number of pharmacological interventions such as corticosteroids and bronchodilators, including beta-agonists. In contrast, other conditions involving breathlessness have received little or no attention and studies continue to focus upon pharmacological approaches. Moreover, although there are a number of non-pharmacological studies that have shown some promise, particularly for COPD, their conclusions are limited by a lack of good quality evidence from RCTs, small sample sizes and limited replication.

**Conclusions:**

More research should focus in the future on the management of breathlessness in respiratory diseases other than asthma and COPD. In addition, pharmacological treatments do not completely manage breathlessness and have an added burden of side effects. It is therefore important to focus more research on promising non-pharmacological interventions.

## Background

Breathlessness is a common symptom which occurs in a wide range of clinical conditions and affects many individuals [1]. Some two-thirds of patients experiencing breathlessness will have a respiratory or cardiac condition [2]. Breathlessness also often occurs in cancer, affecting 50-70% of patients [3]. Both frequency and severity of breathlessness increase towards the end of life [3-5].

The terms breathlessness and dyspnoea are often used interchangeably and definitions focus on sensations of difficulty, discomfort and distress in breathing experienced by those affected [1,3,6], with perhaps the most authoritative definition coming from the American Thoracic Society in 1999:

'...a subjective experience of breathing discomfort that consists of qualitatively distinct sensations that vary in intensity. The experience derives from interactions among multiple physiological, psychological, social, and environmental factors, and may induce secondary physiological and behavioural responses' [1].

The subjective nature of breathlessness has led to comparisons with pain as a symptom [3] -and indeed breathlessness is the most common symptom after pain for which patients seek medical help [2].

Breathlessness can have a significant impact on daily living and health-related quality of life for patients and families [1,3,7-9]. The experience of breathlessness may markedly reduce patients' functional ability [1,3,8], and fear of this symptom can itself lead to activity avoidance [10,11]. Strong associations between breathlessness and fatigue have also been noted [1,12,13], which can further reduce functional ability.

There is a strong relationship between breathlessness and anxiety or panic [7,8,10,11,14], with the latter often resulting in patients who experience breathlessness undergoing emergency hospital admissions. The nature of the relationship between breathlessness and anxiety or panic is complex [1,10] and Bailey's [7] work in chronic obstructive pulmonary disease suggests that anxiety may be a consequence of breathlessness, rather than the reverse. Severe breathlessness may also be associated with depression, again the inter-relationship being complex [14].

Breathlessness is distressing for patients and their families [7,9,15] and may result in a heavy physical, social and emotional burden on carers [3,7,8]. Carers of breathless patients have reported feelings of helplessness, fear, isolation and frustration [3,7,8]. Social isolation is often reported in breathless individuals and their family members [3,8].

Importantly, breathlessness may have prognostic significance. Abidov et al [16] identified increased mortality in patients with known or suspected cardiac disease in whom breathlessness was present, while others [13] comment on the relationship between breathlessness and poor prognosis in non-heart failure populations. Associations between breathlessness and mortality have also been reported in general and elderly population studies [17,18].

Breathlessness is a common and distressing symptom in the advanced stages of both malignant and non-malignant disease. It is characteristic, for example, of chronic obstructive pulmonary disease (COPD), often causing marked impairment in activity [19] and confinement to the home [20]. COPD is a progressive, incurable illness and a leading cause of disability and mortality worldwide [21,22]. Despite this, there is evidence that services for people with COPD are poor and needs (including palliative care needs) are unmet: indeed, breathlessness in COPD has been described as 'invisible', partly because of the lack of attention it receives from health services [23,24]. Breathlessness is also a common and devastating symptom in advanced cancer, with prevalence increasing from referral to palliative care services (15-55.5%) to the last week of life (18-79%) and severity reported to be moderate-severe in 10-63% of cases [25]. It causes major impairment in function and quality of life (QoL) [26]. To date, however, there is limited evidence that palliative care interventions have improved breathlessness or that either medical or nursing care have provided effective assistance to individuals with this symptom [27].

Despite the wide range of conditions that cause distressing levels of breathlessness, including primary and secondary cancer, COPD, cystic fibrosis, interstitial lung disease (ILD) and chronic heart failure (CHF) it is still very difficult to manage [23,28].

## Method

Against this background we conducted an integrative review of pharmacological and non-pharmacological interventions for breathlessness within a wide range of cardiorespiratory diseases. For the purpose of this study, pharmacological interventions were defined as those classified as medicinal products under the EU Directive 2001/83/EEC, i.e. any substance or combination of substances which may be administered to human beings with a view to making a diagnosis or to restoring, correcting or modifying physiological function in human beings. Non-pharmacological interventions are those that are not classified as medicinal products.

This integrative review was undertaken both to provide a clear account of the current body of work on the subject and as a preliminary step in developing further interventions for the management of breathlessness and other respiratory symptoms. Given the large number of systematic reviews available, and the limited clinical utilisation of many of the interventions found (with the exception of a few pharmacological interventions such as steroids and beta-agonists), the decision was taken to focus and limit the integrative review to only systematic reviews.

We searched the following electronic databases for systematic reviews of randomised controlled trials, quasi-experimental trials, and uncontrolled trials where there were group comparisons of both pharmacological and non-pharmacological interventions for breathlessness in non-malignant disease:

• Cochrane Library (Cochrane Airway Review Group)

• AMED (1985-June 2007)

• CINAHL (1982-June 2007)

• EMBASE (1988-June 2007)

• Ovid MEDLINE (1950-June 2007)

• PsycINFO (1985-June 2007)

Searching was carried out with a combination of assigned index (MESH) terms and text words, with truncations where appropriate and different spellings (e.g. dyspnea AND dyspnoea). Between July 2007 and September 2009, while the data of the review was collated and analysed, regular further searches were carried out using the key words in order to identify any new published systematic reviews, adding them to the main body of the current review.

### Databases were searched using the following search strategy

1. asthma

2. COPD

3. Chronic AND obstruction AND pulmonary AND disease

4. 2 or 3

5. Heart AND failure

6. Cystic AND fibrosis

7. interstitial MESH lung disease

8. pulmonary AND arterial AND hypertension

9. bronchiectasis

10. 1 OR 4 OR 5 OR 6 OR 7 OR 8 OR 9

11. dyspnea OR dyspnoea

12. difficult AND breath

13. breathlessness

14. short AND breath

15. short of breath

16. 11 OR 12 OR 13 OR 14 OR 15

17. Systematic AND review

18. 10 AND 16 AND 17

Systematic reviews identified in this way were reviewed independently for inclusion by two of the authors (AM, RD). Any disagreements were resolved by discussion. Many reviews did not explicitly measure breathlessness as an outcome, and for others it was not the primary outcome. For this reason reviews were eligible for inclusion in our review if they reported research on adult participants using not only our primary outcome measure of breathlessness (e.g. Borg and/or modified Borg tests, verbal categorical scales, visual analogue scales), or some other measure of respiratory symptoms (e.g. including cough, wheeze in a composite score), but also if they reported other frequently used breathlessness related outcome measures such as lung function or physiological changes (e.g. spirometry, exercise tolerance, arterial blood gases, pulse oximetry). Although these secondary outcomes may act as proxies for breathlessness, they do have some limitations. For example, lung function only moderately relates to the symptom experience of patients, and exercise tolerance may be as much a consequence of fatigue as breathlessness. Moreover, many reviews also reported 'single symptom scores', which although included several symptoms, among them breathlessness too, yielded only a single composite score. Many reviews also reported upon improvements in other key outcomes (e.g. survival, health-related quality of life) that may be linked or influenced by levels of breathlessness, and these have been included within this review.

Trials of any non-pharmacological intervention were included, as well as any drug/pharmacological intervention (other than radiotherapy and anti-cancer chemotherapy), given by any route or in any dose. Reviews were excluded if they were not available in English, or were wholly or primarily concerned with the treatment of children (i.e. aged < 18 years), infections, sleep apnoea, or cough. They were also excluded if respiratory symptoms were not included as study outcomes.

Data were extracted by one of the authors (RD) using a data extraction form designed for the review and was independently verified by one other author (AM). A third author (AC) had the role of arbitrator in cases of disagreement. Data extracted included the nature of the intervention, the number of trials included in the review, the aggregated sample, primary and secondary outcome measures, the condition treated, and the main conclusions.

Following data extraction, systematic reviews identified were categorised by condition treated (asthma, COPD, bronchiectasis, interstitial lung disease, bronchitis, bronchoconstriction, pulmonary arterial hypertension, breathlessness (generic), and respiratory failure) and by treatment type (i.e. non-pharmacological/pharmacological).

## Results

We initially identified 3359 citations, with an additional 436 resulting from the updated search, giving a total of 3,795 citations. Of these, 3576 were excluded based on the title/abstract as they were irrelevant studies, reviews but not systematic reviews, duplicates, laboratory studies or individual trials which were reported in an identified systematic review. From the 219 systematic reviews identified, 66 were further excluded primarily because they were either paediatric reviews or reviews of medical devices. Of the 153 reviews that met the inclusion criteria, 59 addressed non-pharmacological interventions and 94 addressed pharmacological interventions. A summary of key findings from the review is presented below; further details of individual systematic reviews are set out in the Tables presented in Additional Files 1, 2, 3.

Additional file 1 presents data from systematic reviews of interventions for asthma; Additional file 2 presents data from systematic reviews of interventions for chronic obstructive pulmonary disease (COPD), and Additional file 3 presents data from systematic reviews of interventions for other respiratory diseases.

Within this integrative review we have sought to draw together data from existing systematic reviews. However, few of the papers included meta-analyses. This was due to the quality of some of the studies included, variation of the interventions between and within categories, variability of the reported data and the provision of unreported data. It was therefore not possible, for the purposes of this integrative review, to summarise or draw conclusions regarding effect sizes and other statistical parameters.

### Asthma (non-pharmacological treatments)

A total of 34 systematic reviews were identified. Six were excluded because they referred only to children. Of the remaining 28, 26 were published in the Cochrane Database of Systematic Reviews. Three included data on breathlessness [29-31], while all others assessed breathlessness as part of a composite score of 'asthma symptoms'. In some individual studies these symptom scores used lung function as a proxy for symptoms, others included the Asthma Quality of Life Questionnaire that included symptom categories, while others used the Asthma Symptom Checklist as an outcome. Furthermore, many of the original trials included a 'respiratory symptom' score, of which breathlessness/dyspnoea was only part.

Treatments assessed within the reviews included: acupuncture [32], breathing exercises/training [30,33,34]; dietary measures [35-39]; Heliox [29,31]; homeopathy [40]; control of allergens [41,42]; humidity control/ionisers [43,44]; education programs [45-47]; manual therapy[48]; primary care clinics [49]; psychological interventions [50,51]; individualised care plans [52]; and non-invasive positive pressure ventilation (NIPPV) [53], which enhances ventilation by compensating for fatigued ventilator muscles.

Of those reviews with positive findings, four reported a single study only [35,49,53,54]. Holloway & Ram [34] identified seven randomised or quasi-randomised controlled trials of breathing exercises for asthma, concluding that trends for improvement in QOL were encouraging, although no firm conclusions could be drawn, as only 2 of the 7 studies reviewed showed positive results. Gibson et al [45] concluded from evidence from 12 RCTs of limited (information only) education programs that symptom perceptions may improve, although actual asthma symptoms showed no significant differences. Yorke et al's [50] review of 15 RCTs of psychological interventions was unable to draw firm conclusions, but following pooling of data, reported two trials as showing positive effects on QOL after cognitive behavioural therapy (CBT). Biofeedback also appeared to have a positive effect on peak expiratory flow (PEF) in two studies. Gibson et al's [47] large review of 36 randomised trials concluded that education in asthma self-management involving self-monitoring by PEF or symptoms, together with regular review and written action plans, does improve health outcomes in adults with asthma. While the systematic reviews show that the vast majority of non-pharmacological interventions tested to date have largely been ineffective in relation to asthma symptoms, some effect has been reported in trials of calorie controlled diet [35], inspiratory muscle training [30], manual therapy (particularly massage with relaxation) [48] and psychoeducational interventions [50]. The role of Heliox is unclear, as one review showed no effect on symptoms [29], while another showed that 3 of 15 trials reviewed had a positive effect [31]. Selenium supplementation has shown positive results, but there is only one trial available in the literature that included a small sample of 24 patients [54]. The conclusions of two trials was that there was no current evidence of the effectiveness of the 'Alexander technique' for chronic asthma [55] or that feather bedding and pillows reduced asthma symptoms compared with man-made fibres [56].

### Asthma (pharmacological treatments)

In all, 69 systematic reviews were identified. Eighteen were excluded because they referred only to children, did not specify symptoms as outcomes, or were reviews of studies of vaccines or comparisons between devices. Of the 51 reviews included, 50 were accessed via the Cochrane Database of Systematic Reviews. Seven included data on breathlessness [57-63] while all others assessed breathlessness as part of a single score of 'asthma symptoms'. Again, there was heterogeneity between systematic reviews in the specific outcomes measured within these symptom scores.

Treatments included: anti-leukotrienes [64,65]; beta-agonists (inhaled/non-inhaled) [61-63,66-75]; allergen immunotherapy [76]; anti-IgE [77]; antibiotics [78]; anti-cholinergics [57]; azathioprine [79]; beclomethasone [80-82]; budesonide [83,84]; caffeine [85]; beta-blockers [58]; chloroquine [86]; corticosteroids (inhaled/non-inhaled) [59,60,87-91]; cyclosporine [92]; fluticasone [93-96]; anti-gastro-oesophageal reflux treatment [97]; gold [98]; inhaled/non-inhaled magnesium sulphate [98,100]; macrolides [101,102]; methotrexate [103]; oxatomide [104]; and tailored interventions based on sputum eosinophils [105].

Many reviews report some level of benefit from or equivalence between treatments under review. Clearly steroids (both oral and intramuscular) improve lung function [60], while long-acting beta agonists (LABA) with inhaled corticosteroids (ICS) [68] demonstrate improved outcomes in lung function and symptoms compared to LABA and antileukotrienes, short-acting beta agonists (SABA) [75] or theophylline [74]. Treatments from large scale reviews that report positive effects most strongly include: allergen immunotherapy (57 trials) [76], beclomethasone (60 trials) [82], budesonide (43 trials) [84], fluticasone (75 trials) [96], ICS compared with sodium cromoglycate (25 trials) [88], LABA (67 trials) [62], and LABA vs. SABA (31 trials) [75].

Small reviews of caffeine (6 trials) [85], inhaled magnesium sulphate (6 trials) [99], intravenous magnesium sulphate (7 trials) [100], and tailored interventions based on sputum eosinophils, particularly those with severe asthma and frequent exacerbations (3 trials) [105] also suggested therapeutic benefits. Caffeine (taken orally) has demonstrated a modest, though short-term (up to 4 hours), effect as a bronchodilator.

A number of reviews identified the positive 'steroid sparing' or reduced rescue medication effect of treatments such as Anti-IgE (specifically omalizumab) (14 trials) [88] and LABA [82-84,86] (combined total of 78 trials). One review (67 trials) [62], while reporting the positive steroid sparing effects of LABA, also raised some important safety concerns. These concerns related to a significant increase in asthma related deaths or life threatening experiences for patients treated with LABA, mainly among African-American patients and those not on inhaled corticosteroids. SABA, while generally less effective than LABA, showed in one review (49 trials) [70] that regular use of inhaled SABA can decrease symptoms and rescue medication. A modest effect has been shown with regards to antileukotrienes [64], allergen immunotherapy [76], cyclosporine [91], gold [98], and anticholinergics [57]. However, this modest effect in combination with serious adverse events and/or the need for close monitoring, make therapeutic options such as immunotherapy [74], cyclosporine [92] and gold [98], problematic and inappropriate for clinical use. Two trials found no evidence to indicate that either colchicines [106] or dapsone [107] were effective in the management of steroid dependent asthmatic patients.

### COPD (non-pharmacological treatments)

A total of 28 systematic reviews were identified. One was excluded due to lack of focus on respiratory symptoms. Fifteen of the remaining reviews were published in the Cochrane Database of Systematic Reviews. Twenty also included specific data on breathlessness [108-123], while all others assessed breathlessness as part of a composite COPD 'symptom' score. Treatments included: action plans [124]; oxygen/Heliox [108,110,111,113,121,125]; physical therapy [109]; home care/hospital at home [126,127]; non-invasive positive pressure ventilation (NIPPV)/non-invasive ventilatory support (NIVS) during exercise [119,128]; nutritional supplements [129]; inhalers [130]; rehabilitation [112,131]; education/support [120,132]; inspiratory muscle training [114-116]; physical activity [117]; psychological interventions [118]; and surgery [122].

The majority of reviews (n = 14) were based on fewer than 10 trials: six of these had aggregated samples of more than 150 participants [108-111,119,130]. Positive effects were reported for breathlessness with domiciliary oxygen (6 trials [125]), and short term ambulatory oxygen (31 trials [113]). People with COPD are prone to developing alveolar hypoventilation during exacerbations, which may be contributed to by the administration of high inspired oxygen concentrations. Nevertheless, there was no evidence to indicate whether different oxygen therapies in the pre-hospital setting have an effect on outcome for people with acute exacerbations of COPD [133].

Positive effects were also reported on varied outcomes, including breathlessness, for NIVS during exercise (7 trials) [119], and lung volume reduction surgery for end-stage COPD (19 trials) [122], and for outcomes other than breathlessness for oxygen during training (5 trials) [111] home care/hospital at home (4 trials [126], 7 trials [127]) and individualised action plans (3 trials) [124]. A large review of pulmonary rehabilitation (31 trials) [112] found that it relieves breathlessness and fatigue, improves emotional function, and enhances sense of control; a large review of the chronic care model in COPD (32 trials) [120] found reductions in hospitalization rates and emergency/unscheduled visits, and shorter length of hospital stay, although the result specifically for breathlessness/symptoms was unclear in most trials. Four reviews of inspiratory muscle training concluded that it can improve breathlessness and health-related quality of life in adults with COPD (19 trials [114], 25 trials [115], 15 trials [116], 15 studies [123]) and an important addition to pulmonary rehabilitation (15 trials) [116]. However, limitations have been identified within the literature and Shoemaker et al [123] found that it was unclear whether any improvements were mediated through improved muscle strength and endurance.

Ram et al [134] reviewed 14 trials concerning NIPPV for respiratory failure due to exacerbations of COPD. If applied intermittently and for short periods, NIPPV may be sufficient to reverse respiratory failure, and it was found to have a positive effect on a range of outcomes including mortality, need for intubation, and treatment failure. The authors conclude that trials demonstrate the benefits of NIPPV as first line intervention as an adjunct to usual medical care.

Bausewein et al [28] reviewed non-pharmacological interventions for breathlessness in COPD, cancer, chronic heart failure, interstitial lung disease, and motor neurone disease. Strong evidence was identified to show that neuro-electrical muscle stimulation (NMES) and chest wall vibration (CWV) could relieve breathlessness in COPD, and there was moderate evidence in favour of walking aids in COPD and breathing training in studies of mixed cardio-pulmonary disease and COPD. The authors found only low strength evidence of relief from breathlessness with acupuncture or acupressure, and no evidence for the effect of music. They noted that only a few studies included participants with conditions other than COPD. Insufficient data were available to evaluate evidence for a range of other interventions, including relaxation, counselling and support, case management and psychotherapy.

### COPD (pharmacological treatments)

In all, 22 systematic reviews were identified. Three were excluded (lack of focus on respiratory symptoms/non-intervention studies). Two of the remaining 19 reviews were located from sources other than the Cochrane Database. Sixteen included data on breathlessness [135-149] while the others assessed breathlessness as part of a single symptom score. Treatments included: bronchodilators [135,140,141,144,147,148]; LABA and LABA in addition to steroids [136,142,149]; vaccines [137,139]; inhaled/non-inhaled corticosteroids [143,146,150]; SABA [138,145]; methylxanthines [151]; and mucolytics [152].

Seven reviews were based on evidence from fewer than 10 trials [135,136,138-140,147,151]. One of these referred to an aggregated sample of fewer than 150 participants [138]. In one review the size of the aggregated sample was unclear [148]. Positive conclusions were drawn in respect of some aspect of intervention(s) for COPD in over half of the reviews, including: combined corticosteroid and LABA in a single inhaler (6 trials) [136]; influenza vaccine (6 trials) [137]; ICS (47 trials) [150]; ipratroprium vs. LABA (7 trials) [140]; LABA (23 trials) [142]; mucolytic agents (although their effect was small) (26 trials) [152]; oral corticosteroids (OCS) (24 trials) [143]; oral theophylline with a modest effect on lung function but largely ineffective in relation to breathlessness (20 trials) [144]; SABA (13 trials) [145]; systemic corticosteroids (10 trials) [146]; tiatropium (9 trials) [147]; bronchodilators, i.e. β-agonists, anti-cholinergics and theophyllines (33 trials) [148]; and LABA (12 trials) [149]. A large review of 47 trials (13,139 patients) [150] showed that ICS used for 2-6 months resulted in small symptom improvements, while use for more than 6 months had limited effect on symptoms.

Jennings et al [153] reviewed 18 trials concerning the use of opioids for dyspnoea in both cancer and non-malignant conditions (e.g. chronic heart failure, COPD, interstitial lung disease, pulmonary fibrosis), concluding that opioids have a positive effect on the sensation of breathlessness. Data did not support the use of nebulised morphine in the management of breathlessness.

### Bronchiectasis (non-pharmacological treatments)

Searches identified three systematic reviews. One of these was excluded because it focussed on surgical intervention only. The remaining reviews (both from the Cochrane Database) featured nurse specialist care (1 trial) [154] and physical training (2 trials) [155]. Bradley et al [155] included data on breathlessness. Both reviews were based on aggregated samples of less than 80 participants. French et al [154] reviewed one trial of nurse-led care (no significant changes in outcome measures or significant difference compared to doctor-led care). Bradley et al [156] included two trials of physical therapy, concluding that inspiratory muscle training improved endurance exercise capacity.

### Bronchiectasis (pharmacological treatments)

Searches identified four systematic reviews from the Cochrane database (2 trials [156]; 2 trials [157]; 3 trials [158]; 9 trials [159]) that found evidence of effectiveness. Two reviews [156,157] were based on samples of more than 54; in a third review [158] it was not possible to determine the size of the aggregated sample. One review [156] included data on breathlessness. Wills & Greenstone [156] concluded that dry powder mannitol was shown to improve tracheobronchial clearance in bronchiectasis. Ram et al [157] found that ICS may improve lung function in bronchiectasis, but that larger trials were required to guide practice. Evans et al [159] found a small benefit from prolonged antibiotic use to interrupt the cycle of bacterial colonisation, inflammatory change and progressive lung damage, but also advocated further, more powerful trials. Crockett et al [158] concluded that there was insufficient evidence to evaluate mucolytics for bronchiectasis. There were also seven systematic reviews that found no evidence of effectiveness of pharmacological treatments in the management of bronchiectasis: for oral methylxanthines [160]; LABAs [161]; SABAs [162]; anticholinergic therapy [163]; leukotriene receptor antagonists [164]; oral steroids [165]; and pneumococcal vaccines [166].

### Pulmonary sarcoidosis, pulmonary fibrosis, interstitial lung disease

Five systematic reviews (Cochrane Database) were considered, three of which included data on breathlessness [167-169]. Paramothayan et al [170] reviewed 13 trials of corticosteroids for pulmonary sarcoidosis, concluding that oral steroids may be of use in some circumstances. Paramothayan et al [168] further reviewed five trials and found limited evidence to support the use of immunosuppressive and cytotoxic therapy for this condition. Davis et al [167] reviewed three trials and found little to justify routine use of non-corticosteroid agents for idiopathic pulmonary fibrosis (IPF), and Richeldi et al [171] found no evidence for an effect of corticosteroid treatment in patients with IPF. Polosa et al [169] reviewed a single small trial of nebulised morphine in interstitial lung disease (six participants), which reported no improvement in maximal exercise performance or reduction in breathlessness during exercise.

### Chronic bronchitis

Staykova et al [172] reviewed nine trials of prophylactic antibiotic use in chronic bronchitis/COPD, concluding that this treatment does significantly reduce days of illness due to exacerbations. Routine use is not recommended due to concerns about antibiotic resistance and it is noted that available data are over 30 years old.

### Exercise-induced broncho-constriction

Three reviews were considered from the Cochrane Database [173-175]. Spooner et al [173] (24 trials) compared the effects of inhaled mast-cell stabilisers with single dose SABAs or anticholinergics prior to exercise, concluding that all provided relief against broncho-constriction. Overall, SABAs were more effective than mast-cell stabilisers, which were in turn more effective than anticholinergics. Spooner et al [174] (20 trials) concluded that a single dose of a mast-cell stabiliser (nedocromil sodium) before exercise reduces the severity and duration of exercise-induced bronchoconstriction. Kelly et al [175] (8 trials) found no difference between the effect of nedocromil sodium and sodium cromoglycate on lung function following exercise.

### Pulmonary arterial hypertension (PAH)

Three reviews were considered from the Cochrane Database [176-178]. All included data on breathlessness. Liu & Chen [176] reviewed five trials that evaluated endothelin receptor antagonists (ERAs), finding that, in conjunction with conventional therapy, their use improved Borg dyspnoea scores and exercise capacity amongst patients with idiopathic PAH. Paramothayan et al [177] reviewed nine trials that considered the effects of prostacyclin with conventional therapy, identifying some short-term benefits (e.g. on exercise capacity). Kanthapillai et al [178] reviewed four trials that considered the effects of the vasodilator sildenafil, concluding that the benefits observed in small trials required further validation in more adequately-sized studies.

### Cystic fibrosis

Reid et al [179] reviewed two trials concerning the effect of inspiratory muscle training (IMT) on patients with cystic fibrosis. Individual study results were found to be inconclusive for improvement in inspiratory muscle strength, though one study demonstrated improvement in inspiratory muscle strength endurance. The authors concluded that the benefit of IMT in cystic fibrosis for outcomes in inspiratory muscle strength was supported by weak evidence, but that its impact on exercise capacity, dyspnoea and quality of life was unclear.

## Discussion

Improvements are needed in the management of breathlessness in both malignant and non-malignant conditions. Asthma affects an approximately 5.2 million people in the UK [180], with an estimated 42% of them facing significant challenges in their daily lives due to breathlessness [181]. COPD is projected to rank as the world's third leading cause of death by 2030 [182], while the reported frequency of breathlessness in cancer varies depending upon the stage of disease [183]. As disease becomes advanced, so the incidence of breathlessness tends to increase [3-5]. In advanced cardiorespiratory disease, for example, refractory breathlessness is described as a 'devastating' symptom with few effective palliative options [184]. Solano et al [185] found that breathlessness was one of three 'particularly universal' symptoms (with pain and fatigue) that affected more than 50% of patients with far advanced cancer, AIDS, heart disease, COPD and renal disease. Despite this high prevalence and level of severity, breathlessness and other related symptoms are under-assessed and under-treated [185].

Our review of systematic reviews suggests that while breathlessness in some non-malignant conditions has been intensively addressed, mostly through a small range of mainly pharmacological approaches, there are other conditions that have received little or no attention. The majority of systematic reviews we identified have been conducted on interventions for asthma and COPD. In comparison, no other category of disease produced more than six reviews for pharmacological and non-pharmacological approaches combined.

The emphasis in systematic reviews of pharmacological interventions for asthma is clearly on beta-agonists and corticosteroids, with the use of these for relevant asthma-related conditions and in combination with other appropriate pharmacological agents receiving wide support [59,60,62,69,71-75,88,91,96], though with some safety concerns [62]. Reviews that address the 'steroid-sparing' or rescue medication reduction features of various approaches to the pharmacological treatment of asthma are an important sub-group of studies, given the known side-effect profile of this class of drugs [62,64-66,68,70-73,75,77,82-84,87,88,92,93,96,98,103-105].

Systematic reviews of non-pharmacological interventions for asthma are limited by a lack of good quality evidence from randomised controlled trials. The largest review of non-pharmacological interventions, which assessed the effects of asthma self-management programmes with regular health practitioner review did, however, conclude that this approach, particularly when it includes training to use written action plans to adjust medication, can improve health outcomes in adults with asthma [47].

Bronchodilators, including beta-agonists, are the focus for the majority of reviews of pharmacological interventions for COPD. Positive results were identified among these, and for a number of other agents, including corticosteroids, vaccine, and mucolytics, although balancing potential benefits against known risks remains important. Non-pharmacological interventions with positive outcomes appear to be more established in COPD than in asthma, at least as far as number of trials is concerned. In a number of cases successful interventions were broadly rehabilitative in nature: home care from outreach nursing programmes was shown to have benefits in terms of survival and health related quality of life [127], hospital at home was an effective option for some patients attending emergency departments with an acute exacerbation of their condition [127], pulmonary rehabilitation was found to relieve breathlessness and fatigue, improve emotional function, and enhance sense of control [112], and use of two or more components of the chronic care model resulted in reduced hospitalisations and emergency visits and reduced length of hospital stay [120].

Benefits for a number of outcomes in COPD, including breathlessness, were also found to be provided by IMT within four studies [114-116,124], though one study found that it was unclear whether any such improvement is mediated through improved muscle strength and endurance [123]. Inspiratory muscle training was also found to improve breathing, exercise capacity and quality of life in Bradley et al [155] review of physical training for bronchiectasis, although a similar review in patients with cystic fibrosis showed inconclusive results [179].

In contrast to reviews of asthma treatments, data on breathlessness were included in the majority of reviews of interventions for COPD, both pharmacological and non-pharmacological. Given the distressing nature of breathlessness from the point of view of those who experience it, it is surprising that more studies do not include a specific measure of this symptom. Breathlessness and dyspnoea characterise a range of qualitatively distinct experiences of breathing discomfort, and therefore, outcome measures should arguably take account of patients' perceptions and the language patients use to describe the symptom [186-188]. However, as Bausewein et al [189] point out, no one of the many assessment tools available is comprehensive enough as a single instrument to measure the sensation of breathlessness, its effect on quality of life, and response to treatment. Indeed, this review suggests that the research may emphasise physiological lung changes rather than the subjective experience of a very distressing symptom. It also suggests that findings may reflect lung function rather than symptom experience or symptom distress.

A considerable proportion of the original trials reported in the systematic reviews included either proxy measures for breathlessness (e.g. lung function), used unvalidated measures or used single composite scores for 'respiratory symptoms' that differed both within and between systematic reviews, so providing only indications for the level of improvement related to breathlessness per se. This is quite problematic as the 'validity' and sensitivity of such methods to accurately measure a complex, subjective symptom experience is questionable and can lead to biases, as there is a significant issue with the interpretation of the data, which clinicians often find confusing. Future research should address these measurement issues and use data from validated, patient-reported outcome measures (PROMS) of breathlessness as the primary outcome (e.g. the MRC dyspnoea scale [190] or Dyspnoea-12 [188]), in adequately powered trials.

With the exception of research into treatments for asthma and COPD, the majority of intervention studies, as reflected in our review, continue to focus on pharmacological approaches. We have included two reviews of non-pharmacological interventions for bronchiectasis, but in all other disease categories we found no further examples. More research should focus in the future on the management of breathlessness in respiratory diseases other than asthma and COPD.

As pharmacological treatments do not completely manage breathlessness and they have the added burden of side effects, it seems imperative that non-pharmacological interventions are seen as appropriate complementary interventions to standard pharmacological management, or even an alternative method in selected patients. Despite good evidence from the literature, many non-pharmacological interventions have not become mainstream and are not used in the rehabilitation of patients with respiratory diseases. New ways of thinking about evaluation research, and new combinations of pharmacological and non-pharmacological interventions may be needed for symptoms like breathlessness that continue to impose a heavy burden of distress across a wide range of disease types.

There are also a number of non-pharmacological studies that have shown some promise, although conclusions are difficult due to the small sample sizes they have used and limited replication. Such interventions include oxygen, self-management approaches, breathing exercises, use of fans, acupuncture/acupressure, calorie controlled diet, selenium supplementation, approaches to reduce anxiety and panic, or those interventions that may be theoretically useful but for which no studies have been carried out so far (e.g. Alexander technique or other complementary therapies). Future research should focus on such promising approaches and provide a good evidence-base for practice.

## Conclusions

While the reviews have covered in excess of 2,000 trials, the management of breathlessness remains far from ideal and patients suffer unnecessarily, particularly in the advanced stages of their illness. The development of society guidelines and agreed standards of care should provide a good way for clinicians to navigate across the diversity of potential treatments for breathlessness. Such guidelines should incorporate both pharmacological and non-pharmacological interventions where appropriate. More research is needed in areas of potential benefit, with particular emphasis in the accurate and reliable assessment of breathlessness and in ways that could improve the quality of life as well as health care provision for patients experiencing this distressing symptom. The recent development of clinical guidelines for the management of breathlessness/dyspnea provides some direction for the clinicians in this complex area of symptom management [191].

## Competing interests

The authors declare that they have no competing interests.

## Authors' contributions

Conception of study, development of protocol, study coordination: AM; Searches: RD, RW; Data Extraction, Data Verification, Arbitration: AM, RD, RW, AC, JS; Data Synthesis/Analysis: CB, RW, AM, AC, JS; Data discussions: All authors; Drafting paper: AM, RW, CB. All authors read and approved the final manuscript.

## Pre-publication history

The pre-publication history for this paper can be accessed here:

http://www.biomedcentral.com/1471-2466/10/63/prepub

## Supplementary Material

Additional file 1 **Table 1. Systematic reviews of interventions for asthma**. List of all the studies reviewed under asthma and the data reported in the reviews. Click here for file

Additional file 2 **Table 2. Systematic reviews of interventions for chronic obstructive pulmonary disease (COPD)**. List of all the studies reviewed under COPD and the data reported in the reviews. Click here for file

Additional file 3 **Table 3. Systematic reviews of interventions for other respiratory diseases**. List of all the studies reviewed under other respiratory diseases and the data reported in the reviews. Click here for file
