# Supplementary material for: An integrative review of systematic reviews related to the management of breathlessness in respiratory illnesses
Source: BMC Pulm Med. 2010 Dec 9;10:63. doi: 10.1186/1471-2466-10-63 (PMC3016307; doi:10.1186/1471-2466-10-63)
Supplement: Additional file 1 — Table 1. Systematic reviews of interventions for asthma. List of all the studies reviewed under asthma and the data reported in the reviews. [file 1471-2466-10-63-S1.DOC]

**Table 1: Systematic Reviews of Interventions for Asthma**

| **Authors** | **Technique** | **No. of trials** | **Sample** | **Symptoms** | **Dyspnoea** | **Primary outcomes [measures]** | **Secondary outcomes [measures]** | **Condition**  **/comments** | **Conclusion** |
| --- | --- | --- | --- | --- | --- | --- | --- | --- | --- |
| Reviews with non-pharmacological interventions | | | | | | | | | |
| Rodrigo et al 2006^29^ | Heliox Vs placebo | 10 | 544  Adults/  Children | Yes | Yes | PFT changes (PEFR, FEV1) | Symptoms scores (cough, wheeze, SOB, *dyspnoea*), physiological measures, PaO_2_, SaO_2_), Vital signs | Non-intubated acute asthma patients  Included small studies | Insufficient evidence that Heliox improves the outcomes of patients with severe baseline pulmonary function impairment  No differences in dyspnoea scores |
| Ram et al 2003^30^ | Inspiratory muscle training | 5 | >94  Calculated  adults | Yes | Yes | Inspiratory muscle strength, PFT (FEV1, FVC, PEFR) | Asthma symptoms (*dyspnoea* or breathlessness), Borg score or Visual analogue scale, acute exacerbations | Asthma diagnosis  Limited no. of studies | Insufficient evidence of effectiveness  Significant improvement for maximum inspiratory pressure (PImax) in 3 studies  No data were available for asthma symptoms (dyspnoea) and acute exacerbations |
| Ram et al 2005^33^ | Physical training | 13 | 494  Calculated  adults/  children | Yes | No | Bronchodilator use, symptoms, QOL, Physiological measures  PEFR, FEV1, FVC, VO2max, VEmax, HRmax, MVV, daily diary card (symptoms) | | Asthmatic patients undertaking physical activity | Physical training increases cardiopulmonary fitness and has no effect on worsening of lung function or wheeze  No data about QoL |
| McCarney et al 2003^40^ | Acupuncture | 11 | 324  Adults/  Children | Yes | No | PFT [PEFR, post treatment FEV1, FVC] | QoL exacerbations, symptoms, subjective symptoms | Chronic asthma  Method: variation between studies in types of sham & active acupunctures, outcomes measured, and time points presented | No statistically significant or clinically relevant effects for acupuncture compared to sham techniques  A significant decrease in FEV1% predicted was detected by one study but no differences between the groups (possibly due to placebo effect) |
| Holloway & Ram 2004^34^ | Breathing exercise | 7 | 153  Adults/ Children | Yes | No | Improvement in QoL  No focus on dyspnoea | Symptoms severity, no. of acute exacerbations, Symptom improvement, PFT [PEFR, FEV1, MV] | Asthma diagnosis  Small no. of studies /participants.  Method: studies varied in breathing techniques/outcome measurements | No reliable conclusion  Reduction in rescue bronchodilator use in 2 studies  2 studies show increased QoL |
| Cheng et al 2003^35^ | Calorie controlled diet | 1 | 38 Adults  /Children | Yes | No | Symptoms scores (FEV1 & FVC | PFT [PEFR, FEV1], HRQOL | Chronic asthma | Insufficient evidence of effectiveness  Significant increase in FEV1 & FVC |
| Thien et al 2002^36^ | Dietary marine fatty acids (fish oil) | 9 | 238  calculated  Adult/  Children | Yes | No | PFT (FEV1, PEF), rescue medication use, symptoms, exacerbations, QOL scores, hospitalization | | Asthma diagnosis  Method: supplement (Fish oil) Vs placebo  None of the trials reported asthma exacerbations | No consistent effect of fish oil supplement or a high diet of fish oil in any of the analysed outcomes |
| Ram & Ardern 2004^37^ | Dietary salt reduction or exclusion | 6 | 121  Calculated  Adults/  Children | Yes | No | Symptoms, PFT | Rescue medication use, exacerbations, bronchial hyper-reactivity | Allergic asthma | No significant effect on any asthma outcomes |
| Ram & Ardern 2001^38^ | Tartrazine (food additive) | 6 | 570 calculated  Adults/ Children | Yes | No | PFT (FEV1, PEF), rescue medication use | Symptom scores, non-specific bronchial hyper reactivity to histamine or methacholine, allergen specific bronchial hyper-reactivity | Asthma or allergic asthma  Method: exclusion or challenge (oral administration of tartrazine as a challenge Vs. placebo or dietary avoidance of tartrazine Vs. normal diet) | Limited evidence of effectiveness  Routine tartrazine exclusion may not benefit most patients, except those very few individuals with proven sensitivity |
| Ho et al 2003^31^ | Heliox Vs Air-Oxygen mixtures | 15 | 2285  Adults/  Children | Yes | Yes | PEFR, FVC, PEFR %, RR, FEF25-75, clinical asthma scores, *Dyspnoea Index*/score (DI), PaCO2, length of hospital stay | | Acute asthma | PEFR% improved with Heliox compared to airO2  Significant difference between Heliox and airO2 for *dyspnoea* *index*-data in 3 studies |
| McCarney et al 2004^40^ | Homeopathy | 6 | 556  Adults/  Children | Yes | No | Symptoms in 4 of 6 studies | Lung function (PEFR, FEV1, FVC) (in 5 of 6 studies, QoL, global assessment | Stable chronic asthma  Method: studies varied with regards patients, measures and interventions | No effect on symptom score in any study  Conflicting evidence for pulmonary function |
| Gotzsche et al 2004^41^ | House dust mite control measures | 49 | 2,733  Adults/  Children | Yes | No | Subjective well-being, asthma symptoms scores, PFT (FEV1, PEFR) | | Allergic asthma  Method: physical, chemical or combined methods of reducing mite allergen levels | No significant difference in the number of patients who improved, symptoms scores or medication usage |
| Kilburn et al 2001^42^ | Pet allergen control measures | 2 | 57  Adults/  Children | Yes | No | PFT | Coughing, change in rescue medication use, absence, QoL, symptom scores | Pet allergic asthma  Two small studies only, both examined air filtration units | No significant difference were detected between active intervention and control in all outcome measures |
| Singh et al 2002^43^ | Humidity control | 1 | 40 Adults | Yes | No | PFT (FEV1, PEFR), symptoms, exacerbations, bronchodilators use, QoL, hospitalisation | | Chronic asthma  One study only with low sample size  Method: compared mechanical ventilation with or without high efficiency vacuum cleaners | No clinical benefit to asthma patients |
| Blackhall et al 2003^44^ | Ionisers (negative or positive generators) | 6 | 106  Adults/ Children | Yes | No | PFT (PEF, FEV1), asthma symptoms | HRQOL, Exacerbations, medication use (bronchodilators, ICS) | Chronic asthma  Device effect on symptoms improvement | No significant difference in pulmonary function, symptoms, and medication usage |
| Gibson et al 2002^45^ | Limited (information only) patient education program | 12 | 2,542  Calculated  adults | Yes | No | Asthma admission, PFT, rescue medication use, symptomatic days, perceived disability | | Asthma diagnosis  Education information about: pathophysiology; management of trigger factors; side effects of medication | No improvement in asthma outcomes (asthma symptoms) although perceived symptoms improved in two studies |
| Powell & Gibson 2002^46^ | Self management education | 15 | 2,460  Calculated  adults | Yes | No | Asthma admissions, absenteeism, FEV1, PEF, use of B2 agonists, symptom scores, QOL | | Asthma diagnosis | Self-management using a written action plan based on PEF was found to be equivalent to self-management using a symptoms based written action plan |
| Gibson et al 2002^47^ | Self-management education and regular practitioner review | 36 | 6,090  Calculated  Adults | Yes | No | Hospital admission, absenteeism, FEV1, PEF, rescue medication use, QoL, symptom scores (diary), costs | | Asthma diagnosis  Education, self-monitoring of symptoms, regular review of the treatment by practitioner, written action plan | Self-management education reduced hospitalisation, and improved QoL |
| Hondras et al 2005^48^ | Manual therapy | 3 | 156 Adults/  Children | Yes | No | Lung function (PEFR, FEV1, FVC), QoL, subjective symptoms | | Asthmatic diagnosis  Chiropractic manipulation (two studies), and manipulation massage with relaxation (one study) | Insufficient evidence of effect  No significant difference between chiropractic spinal manipulation & sham manoeuvre  One small trial showed that massage/relaxation improved lung function |
| Ram et al 2005^53^ | Non-invasive positive pressure ventilation (NIPPV) | 1 | 30  Adults | Yes | No | Endotracheal intubation | PFT, length of hospitalization, symptom score (Borg scores, visual analogue scale) | For respiratory failure due to severe exacerbations | Limited evidence of effect  Benefit with NIPPV in FEV1, FVC, and PEFR  No data reported on symptoms |
| Ram et al 2002^49^ | Primary care based clinics | 1 | 195  Adults | Yes | No | Use of secondary care services | Hospital admission, use of primary care services, PFT |  | Limited evidence of effect  Fewer patients in the intervention group were likely to wake at night due to their asthma |
| Yorke et al 2006^50^ | Psychological interventions | 15 | 687  Adults | Yes | No | Health service utilisation | Asthma symptoms, PFT (FEV1, PEFR), medication use, Asthma QoL Questionnaire (AQLQ) | Asthma diagnosis  Behavioural therapy, cognitive therapy, cognitive behavioural therapy (CBT), relaxation, counselling | QoL improved in 2 studies with CBT  PEFR improved by biofeedback |
| Smith et al 2005^51^ | Psycho-educational interventions on health outcomes and costs | 57 | Unclear | Yes | No | Hospital admission, symptom scores | | Severe/ difficult asthma | Some evidence of overall positive effects of psycho-educational interventions on hospital admissions in adults and children, and on symptoms in children |
| Toelle & Ram 2004^52^ | Written individualised management plan | 7 | 967  Calculated  adults/  children | Yes | No | Compliance (to use asthma symptoms & monitoring symptoms) | Altered medication use related to exacerbations, symptom scores (Borg score or VAS), PFT (FEV1, PEF, FVC), exacerbations, hospitalisations, absenteeism | Asthma diagnosis  Written plans were either peak flow or symptoms based, which were compared against each other or to no written plan  Small trials only | No consistent evidence that written plans improved patients outcome |
| Allam & Lucena 2004^54^ | Selenium supplemen-tation | 1 | 24  Adults/  children | Yes | No | Symptom scores | LFT (FEV1, PEFR), selenium level | Chronic asthma  One small study only | Some indication that selenium supplementation may be useful adjunct to asthma medication, in terms of ‘clinical evaluation’  No reported improvement in PFT |
| Ram et al 2004^39^ | Vitamin C supplemen-tation (vs placebo) | 8 | 322  Adults/  children | Yes | No | Symptom scores, PFT (FEV1, PEFR) | QoL, absenteeism, medication use, hospitalisation, exacerbations | Asthma diagnosis | No significant effect on any asthma outcome |
| Dennis & Cates 2000^55^ | Alexander Technique | 0 | No trials met inclusion criteria | Yes | No | Peak flow, PFT (FEV1), reduction in use of medication (both beta2-agonists/ inhaled steroids), QoL, HRQoL days off work and/or school. | | Chronic asthma | No evidence of effectiveness |
| Campbell et al 2000^56^ | Feather vs. Non-feather bedding | 0 | No trials met inclusion criteria | Yes | No | Dust mite allergen levels.  Asthma symptom scores  Med use, No. of unscheduled visits to a physician/hospital, (FEV1, PEFR, provocative concentration that causes a 20% fall in FEV1 (PC20) | | Asthma diagnosis | No evidence of effectiveness |
| Reviews with pharmacological interventions | | | | | | | | | |
| Westby et al 2004^57^ | Anti-cholinergics (Ach/s) | 13 | 205  Adults | Yes | Yes | Asthma symptoms [Daily diaries] | PFT [ PEF], Bronchodilator use, QOL scores, No. of asthma exacerbations | Stable asthma  Two study groups:  1-[Ach Vs placebo], 2-[Ach + SABA Vs SABA alone] | Achs resulted in statistically significant reduction in *dyspnoea* and PEFR compared to placebo (though small clinical significance)  Ach + SABA Vs SABA gave no evidence in respect to symptom scores or PEFR |
| Salpeter et al 2002^58^ | Cardioselective beta-blockers  (IV or PO B-blockers Vs. placebo/other Interventions) | 29 | 381  (?) Adults | Yes | Yes | FEV1, No. of patients reported symptoms, (wheezing, *dyspnoea*, COPD exacerbations), use of SABA | | Reversible airway disease (*asthma*/*COPD*) with revisable bronchial obstruction | These drugs reduce mortality with HTN, HF, & coronary arterial disease  No adverse respiratory effects in short term use (safe), long term safety is still not clear |
| Manser et al 2001^59^ | Corticosteroids | 9 | 344 Adults | Yes | Yes | PFT [PEFR, FEFR, FVC, % predicted of these tests] | symptom scores (*dyspnoea*), physiological measurements [length of stay, vital signs, ABGs, need for intubation] | Acute severe asthma | No differences were identified among the different doses of corticosteroids.  No statistically significant difference in % predicted for FEV1 |
| Rowe et al 2001^60^ | Corticosteroids [IM & oral form] | 7 | Unclear | Yes | Yes | Relapse to additional care | Relapse requiring hospitalization, side effects, B2-agonists use, PFT [PEFR, FEV1], medication use, symptoms scores (cough, SoB, wheeze) | To prevent relapse following acute exacerbations  Sample size was not reported and not implicitly listed in included trials | Significantly fewer patients relapsed to receive additional care, and decreased need for B2-agonist use  No effect on PFT |
| Travers et al 2001^61^ | IV beta2 agonists | 15 | 584 Adults/ children | Yes | Yes | PFT, vital signs, side effects, clinical scores **(**SOB, wheeze in 4 studies; dyspnoea in 1 study) | | Severe acute asthma  Beta2 agonists Vs. placebo or other bronchodilators or ICS | No evidence that the use of IV beta2 agonists is beneficial over alternative regimes or ICS |
| Walters et al 2007^62^ | LABA with or without ICS | 67 | 42,333 Adults/ children | Yes | Yes | Asthma symptoms, cough, SOB, bronchodilator use, exacerbation | Adverse events, reduced use of other asthma medication, QoL | Chronic asthma  Salmetrol used in 50 studies, formoterol used in 17 studies | Improvement in PEF and FEV1, symptoms, rescue medication, QOL (with or without ICS) |
| Richter 2006^63^ | Long and short acting inhaled B2 agonists | Un-clear | Unclear | Yes | Yes | Asthma exacerbation | | Bronchial asthma and COPD  Information obtained from its English abstract only | The benefit-risk–ratios of B2 agonists remains unsettled  Inhaled LABA+ICS improve dyspnoea exacerbations in asthma and particularly in COPD. |
| Ducharme et al 2004^64^ | Addition of Anti-Leukotriene (AL) + inhaled corticosteroids(ICS) | 27 | 5,871 Calculated  adults/ children | Yes | No | No. of patients with exacerbations requiring IV steroids | Changes in: symptom score; QoL; SABA rescue; PFT; hospital admissions | Chronic asthma  AL + ICS Vs. ICS alone | The addition of AL to ICS brings modest improvement to lung function |
| Ducharme & Di Salvio 2004^65^ | Anti-Leukotriene agents | 27 | 8,923 Calculated  adults/  children | Yes | No | No. of exacerbations requiring systematic corticosteroids [Hospital admissions] | Severity of asthma exacerbations, PFT, change in symptom scores, QOL, FEV1 | Recurrent/chronic asthma  Anti-Leukotriene Vs. ICS | In symptomatic adults anti-leukotrienes were less effective (patients were 65% more likely to experience exacerbation requiring systemic steroids);  FEV1, symptoms, QOL improved with ICS |
| Ni Chroinin et al 2004^66^ | Addition of inhaled LABA + ICS | 9 | 1,061 Adults/  children | Yes | No | Exacerbations of moderate intensity [no. of asthma exacerbations] | Hospital admissions, PFT, symptoms scores, QOL, use of rescue SABA | Steroid naïve with persistent asthma  ICS+LABA Vs. ICS alone | No significant reduction in the rate of exacerbations between the combined treatment or ICS alone,  Combined treatment improved lung function and symptom-free days  Insufficient evidence to recommend combined treatment rather than ICS |
| Parames-waran et al 2000^67^ | Addition of IV aminophylline + beta-agonists | 15 | 848 Adults in 14 trials/ calculated | Yes | No | PFT [PEFR, FEV1] | Admission to hospital, vital signs, adverse effects | Acute asthma  Comparing IV aminophyline Vs. placebo, and treated with beta agonists | No significant effect of aminophylline on airflow outcomes, though aminophylline group had higher PEFR |
| Greenstone et al 2005^68^ | Combination of inhaled LABA & ICS Vs. higher dose of ICS | 30 | 9,509 Adults/ children | Yes | No | Rate of patients with asthma exacerbations of moderate intensity | Symptom scores, rate of patients requiring hospitalisation, PFT, measure reflecting chronic asthma control, side effects, QOL, use of rescue SABA | Persistent (recurrent or chronic) asthma | No significant difference between the two regimens (for prevention of exacerbations requiring systemic corticosteroids); but the combination therapy resulted in improvement in lung function, symptoms & use of rescue B2 agonists |
| Camargo et al 2003^69^ | Continuous Vs. intermittent β agonists | 8 | 461 Adults/ children | Yes | No | Change in PFT | Hospital admissions, clinical outcomes [vital signs, symptom scores] | Acute asthma | Continuous β agonists shows significant improvement in PFT |
| Walters et al 2003^70^ | Inhaled SABA | 49 | 7,483 calculated adults/ children | Yes | No | PFT (PEF, FEV1), asthma symptoms scores, rescue bronchodilator use, QOL, asthma exacerbation | | Chronic asthma  Regular Vs. PRN SABA | No clinical or significant difference in pulmonary function  Patients taking regular doses required less rescue medication and had fewer days with asthma symptoms |
| Gibson et al 2005^71^ | LABA as an ICS sparing agent | 10 | 3,011 Calculated  adults/ children | Yes | No | ICS use, clinical outcomes (exacerbations, FEV1, asthma symptoms, rescue medication, side effects) | | Chronic asthma | When using moderate to high doses of ICS, the addition of LABA has an ICS sparing effect |
| Ducharme et al 2006^72^ | LABA Vs. anti-leukotrienes (LTRA) (both when added to ICS) | 11 | 6,030 Adults/Childs | Yes | No | No. of patients with asthma exacerbations | Exacerbations severity (Hospital admission), PFT, symptom scores, QOL | Chronic asthma  Both compared as an odd-on therapy to 400-565 mcg of beclomethasone | Risk of exacerbations was lower with LABA + ICS when compared to LTRA + ICS  LABA + ICS also improved symptoms free days, pulmonary function, rescue medication and patient satisfaction |
| Ni Chroinin et al 2005^73^ | LABA Vs. placebo in addition to ICS | 26 | 8,147  Adults/ children | Yes | No | Asthma exacerbations | Exacerbations severity, PFT, symptoms, QOL, use of rescue SABA | Chronic asthma | The addition of LABA significantly improved FEV1, reduced the rate of exacerbations, increased symptom/rescue-free days, |
| Shah et al 2003^74^ | LABA Vs. theophylline | 12 | 1,329  Adults/ adolescents | Yes | No | FEV1, PEF, side effects, rescue medication use, asthma symptoms | | As maintenance treatment | LABA at least as effective as theophylline in reducing asthma symptoms. |
| Walters et al 2002^75^ | Regular LABA Vs. SABA | 31 | 33,368  Calculated  adults/ children | Yes | No | Asthma symptom scores, bronchodilator use, PEF, QOL, asthma exacerbations rate, side effects | | Stable asthma  Regular treatment | LABA significantly better than SABA for many lung functions (PEF), in lowering asthma symptom scores and rescue meds  No differences on risk of exacerbations |
| Abramson et al 2003^76^ | Allergen immuno-therapy | 57 | 3,506  Adults/ children | Yes | No | Asthma symptoms [scores (28/57)], meds. Required, PFT (16/57) [PEFR, FEV1] | | Asthma diagnosis  Using various forms of allergen specific immunotherapy | Immunotherapy significantly reduces asthma symptoms, use of medication, and improves bronchial hyper-reactivity, but risk of side effects such as anaphylaxis  No consistent effect upon lung function |
| Walker et al. 2006^77^ | Anti-IgE (Omalizumab) | 14 | 3,143 Adults/ children | Yes | No | Steroids intake, asthma exacerbation [hospital admissions] | Asthma symptoms, HRQOL, PFT [FEV1, PEF], side effects | Chronic asthma  Allergic asthma  Omalizumab Vs. placebo | Treatment with IV & SC Omalizumab shows significant reduction of free IgE, ICS use, and exacerbation  Clinical value affected by high costs |
| Graham et al 2001^78^ | Antibiotics | 2 | 97 Adults/ children | Yes | No | Health care utilization (hospital-isation) | PFT, sputum bacteriology, symptom scores, vital signs, treatment cost | Acute asthma  In the 97 patients, 115 exacerbations were reported | No clear conclusion that antibiotic use without evidence of infection is effective |
| Dean et al 2003^79^ | Azathioprine (immuno-suppressive anti-metabolite) | 2 | 23 Adults | Yes | No | Alteration in maintenance oral corticosteroid dose [Medication intake, rescue medication] | PFT [PEF, FEV1, FVC, PaO2] symptoms scores (cough, wheeze), asthma exacerbation[hospitalisation] | Staple steroid dependent asthmatics  Small study sample sizes, methodology shortcomings | Insufficient evidence to determine whether azathioprine is an effective steroid-sparing treatment  No differences in FEV1, FVC, PaO2 and symptoms (wheeze, cough). |
| Adams et al 1999^80^ | Beclo-methasone dipropionate (BDP) | 11 | 1,614  Adults/ children | Yes | No | PFT [FEV1, PEFR], symptoms [scores], rescue beta agonist [medication use], QOL, Asthma exacerbations, adverse effects | | Chronic asthma  Assessing different doses of BDP | Higher doses had significant advantage over lower dose (800 mcg/d over 400mcg/d) and improved PEFR, FEV1, reduced night time symptoms compared to baseline, though questionable clinical significance  No differences in daytime symptoms |
| Adams et al 2000^81^ | Beclo-methasone (BDP) Vs. Budesonide (BUD) | 24 | 1,174 Adults/ children | Yes | No | PFT [FEV1, PEF], symptom scores [diary card], rescue meds. intake, QoL, asthma exacerbations | Adverse effects | Chronic asthma  SR limited by using different delivery devices | No differences between the two drugs on FEV1, morning /evening PEF, asthma symptoms, rescue beta agonists |
| Adams et al 2005^82^ | Beclo-methasone (BDP) Vs placebo | 60 | 6,542 Adults/ children | Yes | No | Airway calibre [FEV1, diary (morn/eve) diary, PEFR] | Symptoms scores, rescue bronchodilators,HRQOL, Asthma exacerbations [hospitalisation] | Inhaled BDP  Chronic asthma | Inhaled BDP can help relieve symptoms of asthma in both children & adults, with improved airflow, reduced bronchodilator use |
| Adams et al 2000^83^ | Budesonide | 24 | 3,907 Adults/ children | Yes | No | Airway calibre [FEV1, morning/evening PEFR], asthma symptoms, rescue SABA, QOL, asthma exacerbations [hospitalization], side effects | | Chronic asthma  Assessing different doses of Budesonide | Significant dose response effect between low and high dose  Improvement in FEV1 (severe asthma), reduced exacerbations (moderate-severe asthma)  No significant dose dependent improvement in FEV1, PEFR or symptoms in non-oral steroids treated mild-moderate asthmatics. |
| Adams et al 1999^84^ | Budesonide Vs placebo | 43 | 2,801 Adults/ children | Yes | No | Airway calibre [FEV1, PEFR (morning/evening)], asthma symptoms, rescue SABA, HRQOL, asthma exacerbations [hospital admission] | | Chronic asthma  SR limited due to variation in methods reporting symptoms | Budesonide is highly effective (improves FEV1, morning & evening PEF)  Doses of 500-800 mcg/d have slightly larger effect sizes than lower doses |
| Bara & Barley 2001^85^ | Caffeine | 6 | 55 Adults | Yes | No | PFT [FEV1, FEV] | Symptoms, side effects, adverse effects, vital signs | Asthma diagnosis  Oral caffeine Vs. placebo | Caffeine (high dose or low) has modest bronchodilator effect (for up to 4 hrs) in asthma, and improves lung function for up to 4 hrs  No information on symptoms |
| Dewey et al 2003^86^ | Chloroquine (anti-inflammatory agent) | 1 | 9 Adults | Yes | No | Steroid consumption | PFT [PEF, FEV1], side effects, symptoms, rescue medication, asthma exacerbations/ hospitalisation | Chronic use of oral corticosteroid  Included only one small study | Insufficient evidence to determine whether Chloroquine is effective as a steroid sparing treatment for asthma  No significant effect on PFT |
| Powell & Gibson 2003^87^ | ICS | 26 | 3,980  Calculated  adults/ children | Yes | No | Asthma symptoms, PFT, exacerbations, unscheduled doctor visit | | High dose ICS Vs. low dose ICS | Low Vs. moderate doses showed no differences in LFT and symptoms  Moderate Vs. High doses showed significant but clinically small improvement in FEV1, not PEF |
| Guevara et al 2006^88^ | ICS Vs. sodium cromoglycate (SCG) | 25 | 1,600 Adults/ children | Yes | No | Asthma exacerbations | LFT, asthma symptoms, bronchodilator use, QoL, side effects | Chronic asthma | ICS showed improved PFT, reduced exacerbations, rescue medication use (bronchodilators) and asthma symptoms greater than SCG  Effect of these medications on HRQOL was limited |
| Edmonds et al 2000^89^ | ICS | 10 | 2,113 Adults/ children | Yes | No | Acute asthma relapse (unscheduled visit) | Asthma specific QoL, PFT, beta-agonist use, symptoms | Acute asthma  ICS (alone or combined with OCS) Vs. OCS  SR limited due to heterogeneity in 2^nd^ outcome measures | No significant difference between the compared regimens in all the measured outcomes (i.e. no benefit of adding ICS to standard OCS)  2 studies tested AQOL and included dyspnoea (though not the concern of the SR) |
| Mash et al 2001^90^ | ICS Vs. OCS | 10 | More than 252  (in 9 of 10 trials)  Persons over 15 years old | Yes | No | Symptoms frequency, PFT (PEF,FEV1, VC), QoL | | Chronic asthma  Included only small studies | A daily dose of 7.5-10 mg/day of prednisolone appears to be equivalent to a mod-high dose of ICS (300-2000 mcg/day)  Side effects may be present, so if no alternative to OCS, the lowest effective dose should be prescribed |
| Taramarcaz & Gibson 2003^91^ | Intranasal corticosteroids (INCS) | 14 | 477 Adults/ children | Yes | No | Symptoms scores (in 10 studies), asthma exacerbations, QOL, PFT (FEV1, PEF), inflammation (eosinophils) | | Coexistent asthma & rhinitis  INCS Vs. placebo or other asthma Treatments | Asthma symptoms scores and FEV1 tended to improve from INCS (though not statistically significant) 4 studies showed this positive impact |
| Evans et al 2000^92^ | Cyclosporine | 3 | 106 Adults | Yes | No | PFT (PEFR, FEV1 and any others) symptoms, rescue medication (bronchodilators), asthma exacerbation (frequency) | | Stable asthma  Cyclosporine as an oral steroid sparing agent | Small but significant treatment effect on steroid dose reductions  One study showed significant improvement in PFT  Questionable clinical significance (serious adverse effects) |
| Adams et al 2005^93^ | Fluticasone (ICS) | 43 | 8,753 Adults/ children | Yes | No | PFT (PEFR, FEV1), asthma symptoms, rescue bronchodilator, HRQOL, rates of asthma exacerbations | | Chronic asthma  Dose escalation | High doses (800-1000μg/day) led to small improvements in PFT compared to low doses (50-100μg/day) in mild-moderate asthma  No effect on symptoms |
| Lasserson et al 2006^94^ | Fluticasone (FP) Vs. HFA beclo-methasone dipropionate (BDA) | 8 | 1,260 Adults/ children | Yes | No | PFT, exacerbations | Symptoms, rescue medication, QOL(AQOL- 4/8 trials), side effects | Chronic asthma | No diff. between FP and HFA-BDA on FEV1 or PFR  This result is only applicable to people who are competent to use metered-dose inhalers. |
| Adams et al 2005^95^ | Fluticasone (FP) Vs. beclo-methasone or budesonide | 57 | 12,614  Adults/  children | Yes | No | PFT (FEV1, PEF) | Symptoms, rescue bronchodilators, HRQOL, asthma exacerbation | Chronic asthma | Fluticasone given at half the daily dose of beclomethasone or budesonide leads to small improvement in PFT  No firm conclusion regarding symptoms |
| Adams et al 2005^96^ | Fluticasone Vs. placebo | 75 | 14,208 Adults/ children | Yes | No | PFT (FEV1, PEF), Symptoms, rescue bronchodilators, HRQOL, asthma exacerbation | | Chronic asthma | Fluticasone is highly effective even in low doses |
| Gibson et al 2003^97^ | Gastro-oesophageal reflux treatment | 12 | 432 Calculated adults/ children | Yes | No | Asthma symptoms, PFT, exacerbations | | Asthma and gastro-oesophageal diagnosis  Treatments: proton pump inhibitors, histamine agonists, surgery, conservative management | Anti-reflux treatment did not improve PFT & asthma symptoms  One trial - (using conservative therapy) reported improvement in experiencing respiratory symptoms (dyspnoea, cough, wheeze, expectoration) |
| Evans et al 2000^98^ | Gold | 3 | 376 Adults | Yes | No | PFT (FEV1, PEF, others), symptoms, rescue meds., frequency of asthma exacerbation, side effects | | Stable asthma  Gold as an OCS sparing agent  2 studies used oral (3mg auranofin twice daily), 1 study used 50 mg IM aurothioglucose | Small but statistically significant treatment effect in terms of steroid dose reduction, but not recommended  Limited clinical significance (due to side effects, necessity for monitoring) |
| Blitz et al 2005^99^ | Inhaled magnesium sulphate (MgSO_4_) | 6 | 296 Adults/ children | Yes | No | Change in PFT from baseline | Clinical severity scores, duration of symptoms, vital signs | Acute asthma  MgSO_4_ (alone or combined with β2 agonists) Vs. β2 agonists | Inhaled MgSO_4_ in addition to β2 agonists improved pulmonary function in patients with severe asthma. |
| Rowe et al 2000^100^ | Magnesium sulphate | 7 | 665 Adults/ children | Yes | No | Admission to hospital – severe attack | PFT (PEFR, FEV1), vital signs, side effects | Acute asthma | IV MgSO4 improved FEV1, PEV in patients with severe acute asthma |
| Richeldi et al 2005^101^ | Macrolides (antibiotics/ anti-inflammatory) | 7 | 416 Adults/ children | Yes | No | Symptom scores, medication use  PFT (FEV1, thoracic gas volume) | Side effects, study withdrawals, eosinophils count | Chronic asthma  Low reporting quality for the studies | Insufficient evidence of effectiveness  4 studies showed a significantly positive effect on symptoms. No significant difference in FEV1 |
| Evans et al 2000^102^ | Troleando-mycine (macrolide antibiotic) as an OCS sparing agent | 3 | 112 Adults | Yes | No | PFT (PEF, FEV1 and others), symptoms, bronchodilators use, asthma exacerbation frequency, QOL scores, change in steroid dosage, side effects | | Steroid dependent asthma  Troleandomycine Vs. placebo | No effect on steroid dose reduction, no benefit in pulmonary functions (in 2 studies) |
| Davies et al 1998^103^ | Methotrexate (MTX) as a steroid sparing agent | 10 | 185 Adults | Yes | No | Alteration in ICS dose, PFT (PEFR, FEV1), symptoms, rescue medication, QOL scores | | Asthma diagnosis | No differences between MTX and placebo for FEV1; MTX shows little relief from asthma but adds side effects |
| Hayashi et al 2003^104^ | Oxatomide (H1-RA) | 6 | 494 Calculated adults/ children | Yes | No | PEF | B2 agonist rescue use, symptoms severity, PFT, QOL, asthma exacerbations, | Stable asthma  Doses varied between trials | No evidence to support oxatomide effect to control patients’ stable asthma  FVC & FEV1 showed significant improvement in 2 studies  No change in symptoms |
| Petsky et al 2007^105^ | Tailored interventions based on sputum eosinophils Vs. clinical symptoms | 3 | 246 Adults | Yes |  | Proportions of asthma exacerbation patients: hospitalisation, QOL and asthma diary, B2 agonists use | Mean clinical improvement (Differences in asthma outcomes) | Asthma diagnosis  Sputum eosinophils strategy Vs. clinical strategy/ symptoms | Tailored asthma interventions based on sputum eosinophils is beneficial in reducing the frequency and severity of asthma exacerbations  Strategy advisable in patients with severe or frequent exacerbations  No differences in clinical symptoms, QOL |
| Dewey et al 2003^106^ | Colchicine (anti-inflammatory agent) | 0 | No trials met inclusion criteria | Yes | No | PEF, FEV1, meds use (e.g bronchodilator) frequency of exacerbation hospitalisations, steroid consumption | | Chronic asthma | No evidence of effectiveness |
| Dewey et al 2002^107^ | Dapsone as OCS sparing agent | 0 | No trials met inclusion criteria | Yes | No | PFT, symptoms, rescue meds, frequency of exacerbations | | Asthma diagnosis | No evidence of effectiveness |
