# Supplementary material for: An integrative review of systematic reviews related to the management of breathlessness in respiratory illnesses
Source: BMC Pulm Med. 2010 Dec 9;10:63. doi: 10.1186/1471-2466-10-63 (PMC3016307; doi:10.1186/1471-2466-10-63)
Supplement: Additional file 2 — Table 2. Systematic reviews of interventions for chronic obstructive pulmonary disease (COPD). List of all the studies reviewed under COPD and the data reported in the reviews. [file 1471-2466-10-63-S2.DOC]

**Table 2: Systematic Reviews of Interventions for chronic Obstructive Pulmonary Disease (COPD**)

| **Authors** | **Technique** | **No. of trials** | **Sample** | **Symptoms** | **Dyspnoea** | **Primary outcomes [measures]** | **Secondary outcomes [measures]** | **Condition**  **/comments** | **Conclusion** |
| --- | --- | --- | --- | --- | --- | --- | --- | --- | --- |
| Reviews with non-pharmacological interventions | | | | | | | | | |
| Ram & Wedzicha 2002^108^ | Ambulatory oxygen | 2 | 70 | Yes | Yes | Exercise capacity | *Dyspnoea*, QOL (CRDQ), PFT, (FVC, MVV, FEV1), PaO2 during exercise, arterial pH, PaCO2 | Stable COPD  Oxygen Vs placebo | No firm conclusion.  One study shows significant effects with reduction of FEV1 and increase in PaO2 at rest. |
| Jones & Rowe 1998^109^ | Bronchopulmonary hygiene physical therapy | 7 | 126 | Yes | Yes | PFT (FVC, FEV1, PEFR), PaO2, Pulmonary Clearance, symptoms (*dyspnoea*), side effects | | Chronic COPD & bronchiectasis  Treatment included chest percussion and postural drainage | Insufficient evidence of effect  No significant effect on pulmonary function, apart from clearing sputum |
| Rodrigo et al 2001^110^ | Heliox | 2 | 66 | Yes | Yes | PFT (PEF, FEV1) | Symptoms scores (wheezing, dyspnoea), PaO2, SaO2, vital signs, side effects, need for mechanical ventilation/ hospital admission | Acute exacerbations  Heliox-O2 Vs air-O2 in non-ventilated, ventilated, or NIPSV | Insufficient evidence to support the use of heliox to treat COPD exacerbations in either ventilated or non ventilated patients  Heliox may make breathing easier (in one study dyspnoea scores (Borg scale) decreased more with heliox than with air-O2) |
| Nonoyama et al 2007^111^ | Oxygen during exercise training | 5 | 105 | Yes | Yes | Maximal exercise capacity, maximum training level achieved during training, end of test Borg dyspnoea scores (3 studies), HRQOL (CRQ), arterial O2 saturation | | COPD diagnosis  O2-supplemented exercise training Vs room air | Limited evidence of effectiveness  Supplemental O2 increased the average exercise time from 6-14min and 6-12min (control)  No significant difference in maximal exercise outcomes, HRQOL or oxygenation status. |
| Lacasse et al 2006^112^ | Pulmonary rehabilitation | 31 | 1,597 calculated | Yes | Yes | HRQOL (9 studies),  Traditional dyspnoea index, SGRQ, CRQ scores) (3 studies), maximal (MEC) or functional (FEC) exercise capacity | | Chronic COPD | Significant improvement found for the 4 CRQ domains improvement was found in all outcomes  No significant effect was detected in symptom scores by CRQ scale |
| Bradley & O’Neil 2005^113^ | Short-term ambulatory oxygen | 31 | 534 | Yes | Yes | Exercise capacity | Dyspnoea scores (Borg or VAS), ABG or pulse oximetry during or post exercise, patient preference | COPD diagnosis/ undergoing exercise test  Limited by small size trials included and publication bias | Ambulatory O2 improved exercise performance in people with moderate –severe COPD  Breathlessness improved SaO2/PaO2 |
| Geddes et al 2005^114^ | Inspiratory muscle training | 19 | 642 | Yes | Yes | Inspiratory muscle strength (P_I_, max) and endurance (inspiratory threshold loading- kPa), exercise capacity (modified Borg scale), work rate maximum (Watts), *dyspnoea* (traditional DI), QOL | | COPD diagnosis  IMT Vs Sham; IMT Vs no intervention; Low Vs high intensity IMT; two different methods of IMT | Targeted resistive or threshold IMT was associated with significant improvement in P_I_, max and kPa, exercise capacity, work rate maximum and *dyspnoea*; while IMT without a target/not using threshold training showed no improvement in these variables  No conclusive evidence regarding QOL |
| Geddes et al 2008^115^ | Inspiratory muscle training | 25 | Unclear | Yes | Yes | Inspiratory muscle strength (P_I_, max) and endurance (inspiratory threshold loading- kPa), exercise capacity (modified Borg scale), work rate maximum (Watts), *dyspnoea* (traditional DI), QOL | | Update of Geddes et al (2005) with six additional studies | Conclusions as above |
| Lotters et al 2002^116^ | Controlled inspiratory muscle training | 15 | 383 | Yes | Yes | PFT, P_I_, max inspiratory muscle endurance, *dyspnoea* rating, 6-or 12-min walking distance, HRQOL | | COPD diagnosis  Inspiratory muscle training (IMT) at an intensity of ≥ 30% P_I_, max  IMT alone or as adjunct to general exercise reconditioning | IMT is an important addition to a pulmonary rehabilitation programme.  Both IMT alone or as an adjunct to general exercise reconditioning significantly increased inspiratory muscle strength and endurance  Significant effect was found for *dyspnoea* at rest & during exercise |
| Chavannes et al 2003^117^ | Physical activity | 5 | 210 | Yes | Yes | Exercise tolerance (cycle ergometry, Walk Test), QOL (CRQ, SGRQ, physiological wellbeing index), *dyspnoea* (VAS, Borg scale, CRQ), number of hospitalisation days, prednisolone courses | | Mild to moderate COPD  Interventions: primary care physiotherapist, home training of large muscle groups, hospital-based muscle training | Physical exercise training can improve the fitness of patients with mild or moderate COPD  4 studies reported positive influence in physical activity  No clear effect on dyspnoea or QOL |
| Rose et al 2002^118^ | Psychologically based treatments to reduce anxiety and panic | 6 | 324 | Yes | Yes | Anxiety, panic, psychological measures (State-Trait Anxiety Inventory-STAI), PFT (FEV1, FVC) | *Breathlessness*, disability, QOL | COPD diagnosis  Emotional distress contributes to loss of function, improvement may be via psychological intervention  A variety of methods were used | Insufficient evidence that psychologically based interventions reduced anxiety in COPD. No study was adequately designed to provide an assessment of psychological intervention aimed at anxiety at COPD. There were no studies of interventions aimed at reducing panic. Two studies (used relaxation, rehabilitation programme) showed significant improvement in dyspnoea |
| Van’t Hul et al 2002^119^ | Non invasive ventilatory support (NIVS) during exercise | 7 | 96 (65 COPD patients) | Yes | Yes | Exertional dyspnoea (Borg scale), exercise endurance (cycling and walking) | | COPD diagnosis  NIVS Vs placebo | NIVS significantly improved *dyspnoea* - NIVS during exercise may reduce exertional dyspnoea and improve exercise endurance |
| Adams et al 2007^120^ | Chronic care model (CCM) | 32 | 5,311 | Yes | Yes | Knowledge, symptoms/ *dyspnoea* (7 RCTs), QOL (10 RCTs-SGRQ), PFT (FEV1 6/20 RCTs), functional status, mortality, health care use (emergency unscheduled visits), performance-based test (Walk test, hospitalisations, length of stay (LoS)) | | Various interventions (self-management support, delivery system design, decision support, clinical information system)  CCM Vs control or comparison group | Dyspnoea score (Borg score in 3 studies) demonstrated statistically significant but clinically insignificant findings. 4 studies reported dyspnoea but showed no significant difference. QOL, lung function and functional status were not significantly different between intervention/ control groups.  Patients who received interventions with 2 or more CCM components had lower rate of hospitalization and emergency/unscheduled visits and a shorter LoS |
| O'Neill et al 2006^121^ | Short-burst O2 therapy | 8 | 159 | Yes | Yes | *Breathlessness* | Exercise capacity, SaO2, O2 consumption, inspiratory capacity, patient preference | Stable COPD  Oxygen for breathlessness at rest, before exercise and after Vs. placebo | Short-burst O2 therapy does not reduce breathlessness before exercise or after exercise, No data for rest state found  Results inconclusive for secondary outcomes |
| Young et al 1999^122^ | Lung volume reduction surgery (LVRS) | 19 | 1,069 | Yes | Yes | Mortality, PFT (FEV1), six minute walking distance (6MWD), QOL (CRQ, the Nottingham Health Profile, SF36), dyspnoea (12 studies) – MRC of Great Britain Scale, CRQ, Borg scale), length of hospital stay, supplemental O2 | | End stage COPD secondary to severe Emphysema  Reduction pneumoplasty, pneumectomy (open or closed procedure, unilateral or bilateral, laser ablation, stapling or both | Fairly consistent results for most outcomes  Physiological improve. in FEV1 appears to be matched by functional improve. in 6 MWD and subjective improve. in dyspnoea and QoL  LVRS appears to represent a promising option |
| Turnock et al 2005^124^ | Action plan | 3 | 320 | Yes | No | Hospital admission, use of medication (antibiotics, steroids) | No./ length of acute exacerbations, QoL, PFT (FEV1) symptom scores | COPD diagnosis  Action plans: guidelines which outline self-initiated interventions | Positive effect on self management knowledge, self-initiation of antibiotics & steroids.  No effect on QoL, lung function, & symptom scores |
| Cranston et al 2005^125^ | Domiciliary oxygen | 6 | 567 Calculated | Yes | No | Survivals, QoL, improvement in physiological parameters | | Chronic COPD | Survival improved with long-term home O2 therapy in patients with severe hypoxemia  In one study O2 reduced exertional dyspnoea  In another trial PaCO2 increased in the control group compared with the O2 group |
| Smith et al 2001^126^ | Home care by outreach nursing | 4 | 624 | Yes | No | PFT (FEV1), HRQOL (SGRQ), hospitalisation, GP visit, nurse visit, carer satisfactions | | Chronic COPD | Significant improvement of HRQOL reported by one trial involving patients with moderate COPD (patients with severe COPD do not appear to benefit)  No change in lung function |
| Ram et al 2003^127^ | Hospital at home | 7 | 754 | Yes | No | Hospitalisation rate, mortality | HRQOL (SGRQ), PFT, exacerbations, bronchodilator use, patient satisfaction, cost | Acute exacerbations  All home support patients had regular visits by nurse at home | Both patients and carers preferred “hospital at home schemes” to inpatient care  1 in 4 carefully selected patients can be safely and successfully treated at home with support from respiratory nurse.  Limited data for QOL and lung function |
| Wijkstra et al 2002^128^ | Nocturnal NIPPV/stable disease | 4 | 86 Calculated | Yes | No | Blood gases, *dyspnoea*, HRQOL, respiratory muscle function, PFT (FEV1, VC), respiratory muscle function | | Stable COPD  Small sample sizes in all studies | Nocturnal NIPPV had no clinically or statistically significant effect on lung function, gas exchange, or respiratory muscle strength |
| Ferreira et al 2005^129^ | Nutritional supplements | 14 | 487 | Yes | No | Anthropometric (body weight., BMI, lean body mass), functional exercise | Lung volumes, respiratory muscle function, peripheral muscle function, HRQOL (2/14) (SGRQ, CRQ) | Stable COPD Supplementations for at least 2 weeks Symptom scores included in QOL scale | Nutritional support had no significant effect on lung function, anthropometric measures or exercise capacity.  No significant difference in CRQ scale.  The results from SGRQ indicated the only domain not significantly favoured by nutritional supplements was symptoms |
| Ram et al 2002^130^ | Pressured metered dose inhalers (pMDI) Vs all other devices | 3 | 61 | Yes | No | PFT (FEV1, FVC, PEFR), QOL | Symptom scores, rescue medication, absenteeism, acute exacerbations | COPD exacerbation  Devices delivered bronchodilators  Stable COPD  Small number of studies | pMDI produced similar outcomes to a dry powdered device for delivering B2 agonists  The soft mist device for ipratropium was more effective than pMDI  Different inhalers might be similarly effective for people with COPD (insufficient conclusion) |
| Salman et al 2003^131^ | Rehabilitation for COPD | 20 | 979 | Yes | Yes | Exercise capacity (walking test), effect on dyspnoea (CRDQ) | | Non-hypoxemia COPD  Meta-analysis | COPD patients who receive rehabilitation have a better exercise capacity and experience less dyspnoea than patients that do not receive rehabilitation |
| Monninkhof et al 2002^132^ | Self-management education | 12 | 1,666  calculated | Yes | No | HRQOL scores, symptoms scores, number & severity of exacerbations, rescue medications, course of oral steroids and antibiotics, days lost from work, lung function and exercise capacity | | Patients with COPD but not asthma | Insufficient data  No effect on day lost from work, hospital admission, lung function  In 2 studies SGRQ showed a better QoL for patients in intervention group |
| Shoemaker et al 2009^123^ | Inspiratory muscle training | 15 | 436  calculated | Yes | Yes | Dyspnoea, HRQOL, exercise tolerance | | COPD | IMT appears to improve dyspnoea, walking test distance, and HRQoL  Numerous limitations within the data |
| Austin & Woodbaker 2006^133^ | Oxygen therapies | 2 | Unclear | Yes | Yes | Mortality from respiratory causes | Dyspnoea score, ABG, hospital stay, PFT | COPD, Acute exacerbation | No data available |
| Ram et al 2004^134^ | NIPPV for respiratory failure | 14 | 778  calculated | Yes | Yes | Treatment failure, mortality, mechanical intubation | Length of hospitalisation, symptom scores/ breathlessness scores (Borg, VAS, verbal rating scores), ABGs | Respiratory failure due to exacerbations of COPD | NIPPV is considered as a first line adjunct therapy to usual medical care for management of respiratory failure due to acute exacerbation of COPD.  Breathlessness was measured by 4/14 trials, from two studies involving 84 patients, Borg scale shows no differences while VAS scale showed signif. improve. In NIPPV |
| Bauswein et al 2008^28^ | Non-pharmacological interventions | 47 | 2532 | Yes | Yes | Visual analogue scales, numerical rating scales, categorical scales, modified Borg scales, disease/ breathlessness specific scales | Domain specific measures for depression/ anxiety, QoL, participant satisfaction, adverse effects, participant withdrawal from studies | Advanced stages of malignant and non-malignant diseases | Breathing training, walking aids, neuro-electrical muscle stimulation and chest wall vibration appear to be effective non-pharmacological interventions for relieving breathlessness in advanced stages of disease |
| Reviews with pharmacological interventions | | | | | | | | | |
| McCrory & Brown 2003^135^ | Anticholinergic bronchodilators (ibratropium bromide (IB)) Vs. B2 agonists | 9 | 525  Calculated | Yes | Yes | PFT (FVC, PEFR, FEV1),ABG (PaO2), symptom scores (*dyspnoea*) | QOL, Functional capacity, health service utilization measure | COPD exacerbation  IB vs beta agonists (4 studies), IB + beta agonists (5 studies) | Comparison: no differences in FEV1 between IB & beta agonists.  IB results in short-term improvement of FEV1 & PEFR.  2 studies reported changes in PaO2 (not significant) |
| Nannini et al 2004^136^ | Combined corticosteroid +LABA (in one inhaler) | 6 | 4,118 | Yes | Yes | Exacerbations (hospital-isation) | PFT (FEV1, FVC), exercise performance (6 min walk), QOL scales, rescue medication, self-rated symptom score/  *breathlessness* | Stable COPD  2 combinations (fluticasone/  salmeterol & Budesonide /formoterol) | Compared to placebo, combination therapy clinically improved exacerbation rates, QOL and lung function.  However, conflicting results when the different combination therapies were compared with the mono-components alone. |
| Poole et al 2006^137^ | Influenza vaccine | 6 | 4,118 | Yes | Yes | No. of acute exacerbations (defined as an increase in *breathlessness* and/or volume and /or purulence of sputum), disability days, hospital admission, mortality, PFT, side effects, cost | | COPD diagnosis  Influenza vaccines Vs placebo | Inactivated vaccine reduced exacerbations in COPD patients  There was significant increase in the adverse reaction but the effects were generally mild and transient |
| Brown et al 2001^138^ | Inhales SABA Vs ipratropium (IB) | 3 | 103 | Yes | Yes | PFT (FEV1, FVC, PEFR), ABG, symptom scores/  Dyspnoea | QOL, functional capacity, hospitalisation, side effects | For treating COPD exacerbation  B2 agonists used (fenoterol and metaproterenol) | Both treatments produced improvement in FEV1 after 90 minutes.  One trial shows improve. in PaO2 with IB compared to metaproterenol.  No data concerning respiratory symptoms |
| Granger et al 2006^139^ | Injectable vaccines | 4 | 941 | Yes | Yes | No. of acute COPD exacerbations (defined as increase in breathlessness and/or volume and /or purulence of sputum) | Pneumonia episodes, hospitalisation, absence, mortality in year following vaccination | For preventing pneumococcal infection in COPD patients | Injectable vaccines do not show that pneumococcal vaccination provides significant protection against disease caused by the bacteria  One trial provided data on acute exacerbations, but no signif. diff. between vaccination and placebo. |
| Appleton et al 2006^140^ | Ipratropium bromide (IB) Vs LABA | 7 | 2,652 | Yes | Yes | PFT (FEV1, FVC, PEFR), HRQOL, *Dyspnoea* scores (at rest or during exercise or self-report), exercise capacity, side effects, rescue bronchodilator use, acute exacerbations | | Stable COPD  IB alone/ combined with LABA Vs LABA alone delivered via pMDI or nebuliser | Monotherapy comparison: significant difference FEV1 & PEFR in favour of salmeterol.  No significant difference in QOL, exacerbations & symptoms (DI)  Combination: significant improvement in lung function, SABA use, and HRQOL compared to salmeterol alone |
| Appleton et al 2006^141^ | Ipratropium bromide (IB) vs SABA | 11 | 3,912 | Yes | Yes | PFT (FEV1, FVC, PEFR), HRQOL (CRQ), *Dyspnoea* scores (at rest or during exercise or self-report), exercise capacity, side effects, rescue bronchodilator use, acute exacerbations | | Stable COPD  IB Vs SABA;  or IB + SABA Vs SABA | The advantage of regular long term use of IB alone or combined with SABA or over SABA alone is small if the aim is to improve PFT, symptoms (dyspnoea scores) and exercise tolerance.  - dyspnoea was measured by one small unblinded trial by Borg score and found to be significant |
| Appleton et al 2006^142^ | Long-acting Beta2 agonists (LABA) | 23 | 6,061 | Yes | Yes | PFT (FEV1, PEFR, FVC), exercise tolerance, QOL (SGRQ, CRDQ), *dyspnoea* (TDI) & symptom scores, incidence of exacerbations, need for rescue meds | | In COPD patients demonstrating poor reversibility to SABA  Salmeterol or formeterol Vs placebo | Salmeterol 50mcg daily compared with placebo reduced exacerbations, lung function and QOL domains of fatigue and dyspnoea |
| Walters et al 2005^143^ | OCS | 24 | 1,142 calculated | Yes | Yes | Change in FEV1 (23 studies), HRQOL (CRQ 3 studies) | Acute exacerbations of COPD, symptoms severity (*breathlessness*, wheeze, dyspnoea) (13 studies), functional capacity and side effects | Moderate-severe COPD or stable COPD | There was significant difference in FEV1 in favour of OCS compared to placebo.  A statistically significant but not clinically significant difference in QOL scores.  No significant difference in symptoms scores except for wheeze. |
| Ram et al. 2002^144^ | Oral Theophylline | 20 | 873  calculated | Yes | Yes | Exercise capacity, PFT (FEV1, FVC), HRQOL scores | ABG (PaO2, PaCO2, SaO2), *Dyspnoea* at rest or during exercise, preferences, acute exacerbations | Stable COPD  Theophylline Vs placebo | Theophylline has a modest effect on FEV1 and FVC and slightly improves PaO2 in moderate to severe COPD  Breathlessness reported in one study (VAS) - no effect found  Wheeze and dyspnoea reported in 2 studies (21 patients) - no effect found |
| Sestini et al 2002^145^ | Short-acting Beta2 agonists (SABA) | 13 | 296  calculated | Yes | Yes | Symptom scores (*dyspnoea* at rest/during exercise, cough, sputum), QOL, PFT (FEV1, PEFR, FVC) | Patient preference, exercise capacity, absenteeism, side effects, mortality | Stable COPD  Breathlessness reported in 6 studies (different measures) | Use of B2-agonists on regular basis for at least seven days is associated with improvement in lung function and decrease breathlessness.  SMD shows highly significant improvement in daily breathlessness scores after treatment with B2-agonists, but not the morning and evening breathlessness scores.  One study found significant improvement in QOL scores for dyspnoea |
| Wood-Baker et al 2005^146^ | Systemic corticosteroids | 10 | 951 | Yes | Yes | Treatment failure (hospital readmission, mortality | PFT, ABG, symptom scores, side effects, *dyspnoea* scores, QOL, functional capacity, sputum production, hospital stay | Acute COPD exacerbations  Parenteral or oral corticosteroids | Oral or parenteral corticosteroids significantly reduced treatment failure. It increased rate of improvement in lung function (FEV1) and dyspnoea and blood gases over the first 72 hours, but significantly increased risk of adverse reactions |
| Barr et al 2005^147^ | Tiotropium (anti-cholinergic) | 9 | 6,584 | Yes | Yes | Exacerbations, hospitalisation, mortality | HRQOL, self-related symptom scores/*breathlessness* (DI), change in FEV1, FVC, exercise performance, inhaled rescue meds, side effects | Stable COPD Tiotropium Vs placebo or LABA  For >one month | Tiotropium reduced COPD exacerbations and related hospitalisations compared to placebo and ipratropium  - It improved HRQOL (SGRQ) and symptom scores (DI) among patients with modest and severe disease and may have slow decline in FEV1 |
| Liesker et al 2002^148^ | Broncho-dilators | 33 | Review included 3 major pharma-cological treatments | Yes | Yes | *Dyspnoea*, steady-state capacity, incremental exercise capacity | | COPD diagnosis  The effect of bronchodilator (B2-agonists, anticholinergics and theophylline) on exercise capacity | Ach/s agents have significantly beneficial effects in the majority of studies  SABA have favourable effects on exercise capacity in more than two third of the studies.  The studies reporting dyspnoea found improvement even in the absence of improvement in exercise capacity |
| Husereau et al 2004^149^ | LABA | 12 | 2,906 | Yes | Yes | PFT (FEV1, PEFR), exercise capacity, QOL, *dyspnoea* (symptom diary scores), COPD exacerbations, rescue use of Sabutamol | | Poorly reversible COPD  LABA Vs placebo | 11 studies reported improvement in FEV1  6 studies reported less rescue inhaler use,  5 studies reported improved dyspnoea scores.  No clinical advantages of salmeterol and formeterol over ipratropium (a less expensive drug) found |
| Yang et al 2007^150^ | ICS | 47 | 13,139 | Yes | No | PFT | Mortality, exacerbations of COPD, QOL (SGRQ) & symptom scores, exercise capacity, rescue bronchodilators | Stable COPD  Compare any dose of any type of ICS with placebo | Medium use of ICS (> 2 – 6 months) resulted in small improve. in FEV1,  Long term use (>6 months) did not significantly reduce the rate of decline in FEV1 and reduced exacerbation rate.  ICS slowed the rate of decline in QOL but increased risk of oropharyngeal candidiasis and hoarseness |
| Barr et al 2003^151^ | Methyl-xanthines | 4 | 169 | Yes | No | PFT (FEV1), hospitalisation, length of hospital stay, self-rated symptoms scores, complication, side effects | | Acute COPD exacerbations  Methylxanthine Vs placebo | Data on clinical outcomes were sparse.  Methylxanthine should not be used for COPD exacerbations |
| Poole & Black 2006^152^ | Mucolytic agents | 26 | 7,335 | Yes | No | No. of acute exacerbations, no. of pts with exacerbations, no. of days of disability (off work) | LFT (FEV1, FVC, PEFR), side effects of treatment | Stable chronic bronchitis or COPD  Acute exacerbations (defined as increase in cough and/or purulent of sputum) | Treatment with mucolytic was associated with small reduction in acute exacerbations and total no. of days of disability.  No difference in lung function |
| Jennings et al 2002^153^ | Opioids | 18 | 271 | Yes | Yes | Dyspnoea (VAS, Borg score, likert scale), O2 cost diagram | | Dyspnoea (any cause)  Oral and parental opioids | Stat. Signif. positive effect of opioids on the sensation of breathlessness (p=.0008)  Inconclusive result for nebulised opioids (no benefits over nebulised O2) |
