# Supplementary material for: An integrative review of systematic reviews related to the management of breathlessness in respiratory illnesses
Source: BMC Pulm Med. 2010 Dec 9;10:63. doi: 10.1186/1471-2466-10-63 (PMC3016307; doi:10.1186/1471-2466-10-63)
Supplement: Additional file 3 — Table 3. Systematic reviews of interventions for other respiratory diseases. List of all the studies reviewed under other respiratory diseases and the data reported in the reviews. [file 1471-2466-10-63-S3.DOC]

Table 3: Systematic Reviews of interventions for other respiratory diseases

| **Authors** | **Technique** | **No. of trials** | **Sample** | **Symptoms** | **Dyspnoea** | **Primary outcomes [measures]** | **Secondary outcomes [measures]** | **Condition**  **/comments** | **Conclusion** |
| --- | --- | --- | --- | --- | --- | --- | --- | --- | --- |
| Reviews with non-pharmacological interventions | | | | | | | | | |
| Bradley et al 2002^155^ | Physical training | 2 | 52 Adults/ children | Yes | Yes | Exercise tolerance | Respiratory muscle strengths (PImax, PEmax), PFT (FVC, PEFR, TLC), *dyspnoea*, QOL (CRQ), expectorated secretions, change in physical symptoms (cough, wheeze), acute exacerbations, compliance, cost | Bronchiectasis`  Two studies published in abstract used IMT  IMT Vs sham or no IMT | IMT improved endurance exercise capacity, maximum inspiratory pressure and QOL  Data represent IMT and not other physical training techniques including pulmonary rehabilitation |
| French et al 2003^154^ | Nurse specialist care | 1 | 80 Adults/ children | Yes | No | PFT, exacerbations | Patient satisfaction, QOL, hospital admission, cost effectiveness, compliance, exercise capacity | Bronchiectasis  Nurse led care Vs traditional model of physician directed treatment | Limited evidence of effectiveness  No significant difference between nurse led care and doctor led care in terms of lung function or QOL, but significantly increased costs in nurse led care due to hospital admission and use of IV antibiotics |
| Reid et al 2008^179^ | IMT | 2 | 45 | Yes | Yes | FEV1; FEVC | Dyspnoea | Cystic fibrosis | The benefit of IMT in adolescents and adults with cystic fibrosis is supported with weak evidence |
| Reviews with pharmacological interventions | | | | | | | | | |
| Wills & Greenstone 2006^156^ | Inhaled hyperosmolar agents | 2 | 28 Adults/ children | Yes | Yes | Symptoms (cough, sputum volume and ease of exacerbation, wheeze, *dyspnoea*), PFT, QOL (SF36, SGRQ), frequency and duration of exacerbations, hospitalisation, side effects, mortality | | Non-cystic fibrosis bronchiectasis | Inhalation of dry powder Mannitol and of hypertonic saline solution can help clear lung secretions in bronchiectasis.  More research is needed |
| Ram et al 2000^157^ | Inhaled steroids | 2 | 54 Adults/ children | Yes | No | Symptoms (daily sputum production, symptom scores), change in PFT (PEFR, FEV1), use of antibiotics | | Non-cystic fibrosis bronchiectasis | Regular use of ICS may improve lung function and inflammatory indices but not symptoms.  Large studies are required |
| Crockett et al 2001^158^ | Mucolytics | 3 | Unclear | Yes | No | PFT (FEV1, PEFR, FVC), hospitalisation rate, QOL, relapse rate and duration of exacerbations | | Non-cystic fibrosis bronchiectasis  Ingested or inhaled mucolytics Vs placebo | Insufficient evidence off effectiveness  High doses of Bromhexine with antibiotics eased difficulty in expectoration, and reduced sputum production  Compared to placebo, recombinant human DNase showed no difference in FEV1 or FVC and increased influenza-like symptoms |
| Evans et al 2007^159^ | Prolonged antibiotics | 9 | 378 Adults/ children | Yes | No | Sputum diary cards, sputum volume and purulence, bacterial colonisation, PFT (FEV1), infection markers (leukocyte count, ESR), acute exacerbations, hospitalisation | | Bronchiectasis  Antibiotics Vs placebo | Small advantage occurs (response rate)  No significant effect on exacerbations and lung function  Antibiotic resistant remains a concern |
| Steele et al 2000^160^ | Oral methyl-xanthines | 0 | No trials met inclusion criteria | Yes | Yes | Symptoms change: breathlessness, cough, sputum vol and purulence; FEV1 and peak flow; No. and severity of acute exacerbations and hospitalisation, QoL | | Bronchiectasis | No evidence of effectiveness |
| Sheikh et al 2001^161^ | LABA | 0 | No trials met inclusion criteria | Yes | No | Respiratory symptom scores; PEF, FEV1, FVC; rates of admission and lengths of stay, exacerbations, QoL, mortality | | Bronchiectasis | No evidence of effectiveness |
| Franco et al 2003^162^ | SABA | 0 | No trials met inclusion criteria | Yes | No | FEV1, PEF, symptom scores, QoL | Sputum volumes; No. exacerbations/ hospitalisations | Bronchiectasis | No evidence of effectiveness |
| Lasserson et al 2001^163^ | Anti-cholinergics | 0 | No trials met inclusion criteria | Yes | Yes | No. of exacerbations | Symptoms (eg sputum volume, dyspnoea); QoL; FEV1 | Bronchiectasis | No evidence of effectiveness |
| Corless & Warburton 2000^164^ | Leukotriene receptor antagonists | 0 | No trials met inclusion criteria | Yes | No | Symptom scores, FEV1, FVC, QoL, exacerbation rates, mortality | | Bronchiectasis | No evidence of effectiveness |
| Lasserson et al 2001^165^ | Oral steroids | 0 | No trials met inclusion criteria | Yes | Yes | Symptoms (e.g. shortness of breath); QoL; FEV1; inflammation markers; morbidity; mortality | | Bronchiectasis | No evidence of effectiveness |
| Chang et al 2007^166^ | Pnumococcal vaccines | 0 | No trials met inclusion criteria | Yes | No | Respiratory exacerbations; hospitalisations; symptom scores | | Bronchiectasis | No evidence of effectiveness |
| Davies et al 2003^167^ | Immuno-modulatory agents | 5 | 141 Adults | Yes | Yes | Mortality, symptom scores (*dyspnoea* at rest/on exercise, cough), exercise capacity, QOL, ABG, hospitalisation, PFT (FEV1, PEF, FVC) | Side effects, patient preference, study withdrawals | Idiopathic pulmonary fibrosis  Different agents were used: azathioprine, colchicine, interferon-gamma | Little good quality information  No evidence to support the use of anti-inflammatory drugs in IPF  Only interferon was shown to produce significant improvement in pulmonary function and arterial oxygenation |
| Paramothayan et al 2006^168^ | Immuno-suppressive and cytotoxic therapy | 5 | 164  Calculated  adults | Yes | Yes | PFT, change in x-ray, symptom scores (*dyspnoea*), mortality, s.e, steroid usage | | Pulmonary sarcoidosis  Methotrexate, chloroquine, cyclosporine A, pentoxifylline | Data on lung function, chest x-ray scores and dyspnoea were largely inconclusive  All drugs were associated with side effects |
| Polosa et al 2002^169^ | Nebulised morphine | 1 | 6 Adults | Yes | Yes | Dyspnoea scores, HRQOL, exercise capacity, PFT (FEV1, VC, TLC), cough counts/scores, ABG, side effects, mortality | | Severe ILD  One small RCT only  Morphine Vs placebo (N/S) | Nebulised morphine did not improve maximal exercise performance and did not reduce dyspnoea during exercise |
| Paramothayan et al 2005^170^ | Corticosteroids | 13 | 1,066 | Yes | No | Change in chest X-ray, PFT (FEV1, FVC) | Symptom scores, side effects | Sarcoidosis | Oral steroid dose was equivalent to prednisolone 4-40 mg/day  Some short term benefit in chest x-ray  In one study symptoms improved at the end of six months of treatment |
| Staykova et al 2001^172^ | Prophylactic antibiotics | 9 | 1,055 | Yes | No | The number of acute exacerbation (increased in cough & in sputum volume) | The duration of exacerbations (days of disability), additional antibiotics required, side effects | Chronic bronchitis | Included trials are over 30 years old [antibiotics sensitivity and discovery of new antibiotics] |
| Spooner et al 2003^173^ | Mast cell stabilising agents in EIB | 24 | 518 Adults/  children | Yes | No | % fall in PFT | % of pts who received complete protection from EIB, No. of pts who received clinical protection, side effects, sub. outcomes (symptom scores/prefer) | Exercise induced bronchoconstriction (EIB)  Nedocromil sodium or sodium cromoglycate or anti-ch (atropine or ipratropium bromide) | All drugs were effective at attenuating the exercise-induced bronchoconstriction response but to varying degrees even in the same individual  Mast cell stabilisers more effective than anti-ch at attenuating bronchoconstriction |
| Spooner et al 2002^174^ | Nedocromil sodium for preventing Exercise-induced bronchoconstriction (EIB) | 20 | 280 Adults/ children | Yes | No | % fall in PFT (FEV1, PEFR), mean % protection | Symptom scores, physiologic measures, performance measures, s.e | EIB  Nedocromil sodium Vs. placebo | Nedocromil sodium (4mg inhaled 15-60 min prior to exercise) sig. reduced severity and duration of EIB when compared to placebo FEV1 improved, time to recover normal lung function shortened No adverse effects with short term use |
| Kelly et al 2000^175^ | Nedocromil sodium Vs sodium cromoglycate | 8 | 117 Adults/ children | Yes | No | PFT (FEV1, PEFR) | No. of participants who received clinical protection, side effects | EIB | No significant difference noted between NGS and SCG with respect to the minimum percent decrease in FEV1 |
| Liu & Chen 2006^176^ | Endothelin receptor antagonists (potent vasodilators) | 5 | 482 Adults/ children | Yes | Yes | Exercise capacity (6-min walk), Borg *dyspnoea* scores, mortality | Cardiopulmonary haemo-dynamics (mean pulmonary artery pressure, pulmonary vascular resistant, cardiac output), PFT | Pulmonary hypertensions (PH) | Endothelin receptor antagonists in conjunction with conventional therapy can improve exercise capacity, Borg dyspnoea scores and several cardiopulmonary haemodynamics |
| Paramothayan et al 2005^177^ | Prostacyclin | 9 | 1,175 Adults | Yes | Yes | Exercise capacity (6-min walk), improvement in NYHA functional status | Cardiopulmonary haemo-dynamics (mean pulmonary artery pressure, pulmonary vascular resistant, cardiac output), Borg/ symptoms of pulmonary hyper-tension, PFT, mortality | Pulmonary hypertension  IV prostacyclin Vs. usual care; oral prostacylin or inhaled Vs. Placebo; SC treprostinil Vs. placebo | Prostacyclin may benefit patients with Pulmonary hypertension.  There is evidence that IV prostacyclin in addition to conventional therapy can confer some short-term benefits in exercise capacity and cardiopulmonary haemodynamics |
| Kanthapillai et al 2004^178^ | Sildenafil (Viagra) | 4 | 77 Adults | Yes | Yes | Improvement in NYHA functional status | Cardiopulmonary haemodynamics (PAP, CO), ABG, exercise capacity, side effects, HRQOL, *dyspnoea* scores, mortality, hospitalisation | Pulmonary hypertension | Sildenafil has a pulmonary vasodilator effect and improved in symptoms  Longer term effects on lung function, symptoms, mortality and exercise capacity require further validation |
| Jennings et al 2007^153^ | Opioids | 18 | 271 | Yes | Yes | *Dyspnoea* (VAS, Borg score, likert scale), O2 cost diagram | | Dyspnoea/ any cause  Oral and parenteral opioids | A significant effect of opioids found on the sensation of breathlessness (p=0.0008)  Inconclusive result for nebulised opioids efficacy (results show there is no benefits over nebulised normal saline) |
| Richeldi et al 2003^171^ | Corticosteroids | 0 | No trials met inclusion criteria |  |  | Mortality | Symptom scores (eg dyspnoea at rest/ on exertion); QoL; ABG; Lung function (FEV1, PEF, FVC) |  |  |
